# Impact of Endothelial Lipase on Cholesterol Efflux Capacity of Serum and High-density Lipoprotein

**DOI:** 10.1038/s41598-017-12882-7

**Published:** 2017-10-02

**Authors:** Irene Schilcher, Sabine Kern, Andelko Hrzenjak, Thomas O. Eichmann, Tatjana Stojakovic, Hubert Scharnagl, Madalina Duta-Mare, Dagmar Kratky, Gunther Marsche, Saša Frank

**Affiliations:** 1 Institute of Molecular Biology and Biochemistry, Center of Molecular Medicine, Medical University Graz, Neue Stiftingtalstraße 6/6, 8010 Graz, Austria; 2 Institute of Experimental and Clinical Pharmacology, Medical University of Graz, Universitätsplatz 4, 8010 Graz, Austria; 3 Division of Pulmonology, Department of Internal Medicine, Medical University of Graz, Auenbruggerplatz 20, 8036 Graz, Austria; 4 Ludwig Boltzmann Institute for Lung Vascular Research, Stiftingtalstrasse 24, 8010 Graz, Austria; 5 Institute of Molecular Biosciences, University of Graz, Heinrichstrasse 31, 8010 Graz, Austria; 6 Institute of Medical and Chemical Laboratory Diagnostics, Medical University of Graz, Auenbruggerplatz 15, 8036 Graz, Austria; 7 BioTechMed-Graz, Graz, Austria

## Abstract

Endothelial lipase (EL) is a potent modulator of the structural and functional properties of HDL. Impact of EL on cholesterol efflux capacity (CEC) of serum and isolated HDL is not well understood and apparently contradictory data were published. Here, we systematically examined the impact of EL on composition and CEC of serum and isolated HDL, *in vitro* and *in vivo*, using EL-overexpressing cells and EL-overexpressing mice. CEC was examined in a validated assay using ^3^H-cholesterol labelled J774 macrophages. *In vitro* EL-modification of serum resulted in complex alterations, including enrichment of serum with lipid-free/-poor apoA-I, decreased size of human (but not mouse) HDL and altered HDL lipid composition. EL-modification of serum increased CEC, in line with increased lipid-free/-poor apoA-I formation. In contrast, CEC of isolated HDL was decreased likely through altered lipid composition. In contrast to *in vitro* results, EL-overexpression in mice markedly decreased HDL-cholesterol and apolipoprotein A-I serum levels associated with a decreased CEC of serum. HDL lipid composition was altered, but HDL particle size and CEC were not affected. Our study highlights the multiple and complex effects of EL on HDL composition and function and may help to clarify the seemingly contradictory data found in published articles.

## Introduction

The best-studied atheroprotective activity of high-density lipoprotein (HDL) is the promotion of reverse cholesterol transport (RCT). RCT is a dynamic process by which HDL removes excess of the peripheral cholesterol for delivery back to the liver for excretion^1^. The first and a key step in RCT is HDL-mediated cholesterol efflux, a process by which HDL removes excess cholesterol from foam cell macrophages in the artery wall^2^.

Among various cellular and plasma factors, endothelial lipase (EL) has been shown to be a strong negative regulator of HDL plasma levels^3–5^ and a potent modulator of the structural and functional properties of HDL^5–7^.

EL is a member of the triacylglycerol (TAG) lipase gene family^8,9^. A distinct feature of EL compared with other family members is its endothelial expression. Following intracellular maturation, EL is secreted as a 68 kDa glycoprotein, a portion of which is cleaved and inactivated by the members of mammalian proprotein convertases^10^. While by virtue of its bridging function EL facilitates HDL particle binding and uptake, as well as the selective uptake of HDL cholesteryl esters^11^, by its phospholipase activity EL cleaves HDL-phospholipids liberating fatty acids and lysophospholipids, which are efficiently taken up by EL-expressing cells^12^. EL is a negative regulator of HDL plasma levels exemplified by increased HDL levels in mice lacking functional EL or decreased HDL levels upon EL overexpression^3^. However, the role of EL in atherosclerosis still remains inconclusive: in one study using apolipoprotein E (apoE)-deficient mice it has been shown that EL deficiency attenuates the progression of atherosclerosis^13^, whereas in another study EL had no impact on atherosclerosis development in apoE- or LDL receptor-deficient mice^14^. Similarly inconclusive are the results from studies addressing the impact of EL overexpression on cholesterol efflux capacity (CEC) of mouse serum; while in one study the adenovirus-mediated EL overexpression in human apolipoprotein (apo) AI transgenic mice markedly augmented the CEC of serum^15^, exactly the opposite was found in mice in which EL overexpression was achieved by profurin-overexpression-mediated furin inhibition^16^. In humans, genetic inactivation of EL resulted in increased HDL cholesterol levels and increased CEC of apolipoprotein B-depleted serum (apoB-DS)^5^.

Because of the inconclusive data from literature and the lack of data on the impact of EL overexpression on the CEC of human serum, we studied the impact of EL on CEC of serum, apoB-DS and HDL generated *in vitro* and *in vivo*.

## Results

### *In vitro* EL-modification of human serum augments CEC of serum but decreases that of isolated HDL

**Figure 1:**
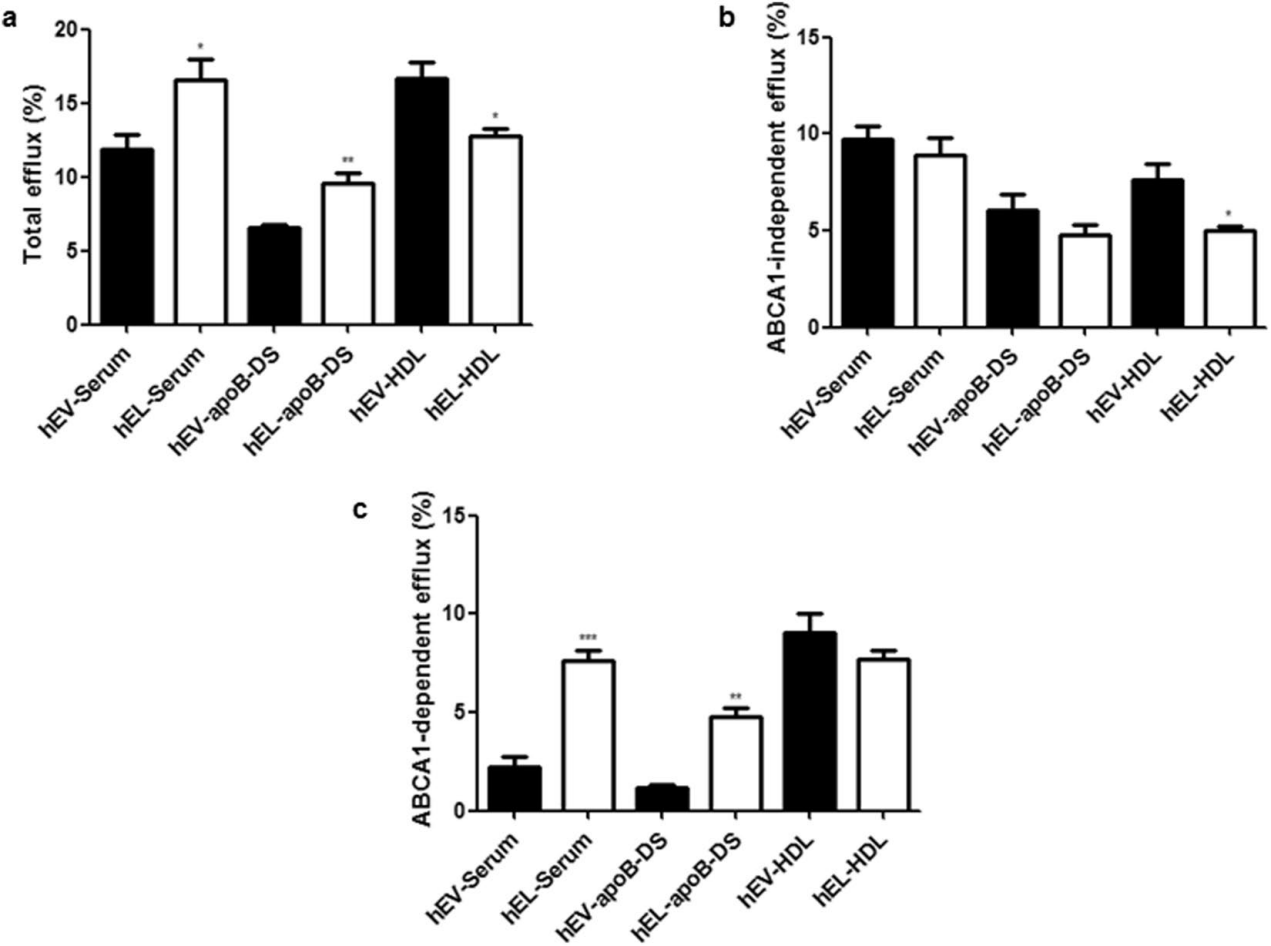
Cholesterol efflux capacity of human (h) EL-serum, hEL-apoB-DS and hEL-HDL. (**a**) Total CEC was measured in J774 macrophages following induction of ABCA1 expression. (**b**) ABCA1-independent efflux was measured without ABCA1 upregulation and (**c**) ABCA1-dependent CEC was obtained by subtraction of the ABCA1-independent efflux from the total efflux. The cholesterol efflux was expressed as the radioactivity in the medium relative to total radioactivity in the medium and cells. Results are mean ± SEM of 5 independent modifications of pool-serum, each measured in duplicates and analysed by two-tailed unpaired t-test. *P < 0.05, **P < 0.01, ***P < 0.001.

Human (h) serum was modified with HepG2 cells overexpressing human EL or with empty virus (EV)-transduced control HepG2 cells (Supplementary Fig. S1) followed by measurements of CEC of serum (both total serum as well as apoB-DS) and isolated HDL in ^3^H-cholesterol labeled J774 macrophages under basal conditions or following ABCA1 upregulation. Total CEC of both hEL-serum and hEL-apoB-DS (Fig. 1a) was significantly higher (p* = *0.032 and p = 0.008, respectively) compared to the respective hEV-controls. In contrast, CEC of hEL-HDL, isolated from the hEL-modified serum, was significantly lower (p* = *0.020) compared to hEV-HDL (Fig. 1a). While ABCA1-independent efflux of both hEL-serum and hEL-apoB-DS was similar to that of their respective hEV-controls (Fig. 1b), the ABCA1-dependent efflux of hEL-serum and hEL-apoB-DS was significantly higher (p* = *0.0003 and p = 0.0011, respectively), compared to the respective hEV-controls (Fig. 1c). The ABCA1-independent CEC of hEL-HDL was significantly lower (p = 0.027) and the ABCA1-dependent efflux was not significantly altered, compared to hEV-HDL (Fig. 1b and c, respectively). From these results we conclude that a newly formed component of hEL-modified serum, but not mature HDL, is responsible for its increased CEC.

### Modification of human serum with EL-overexpressing cells decreases HDL size and generates lipid-free/-poor apoA-I

**Figure 2:**
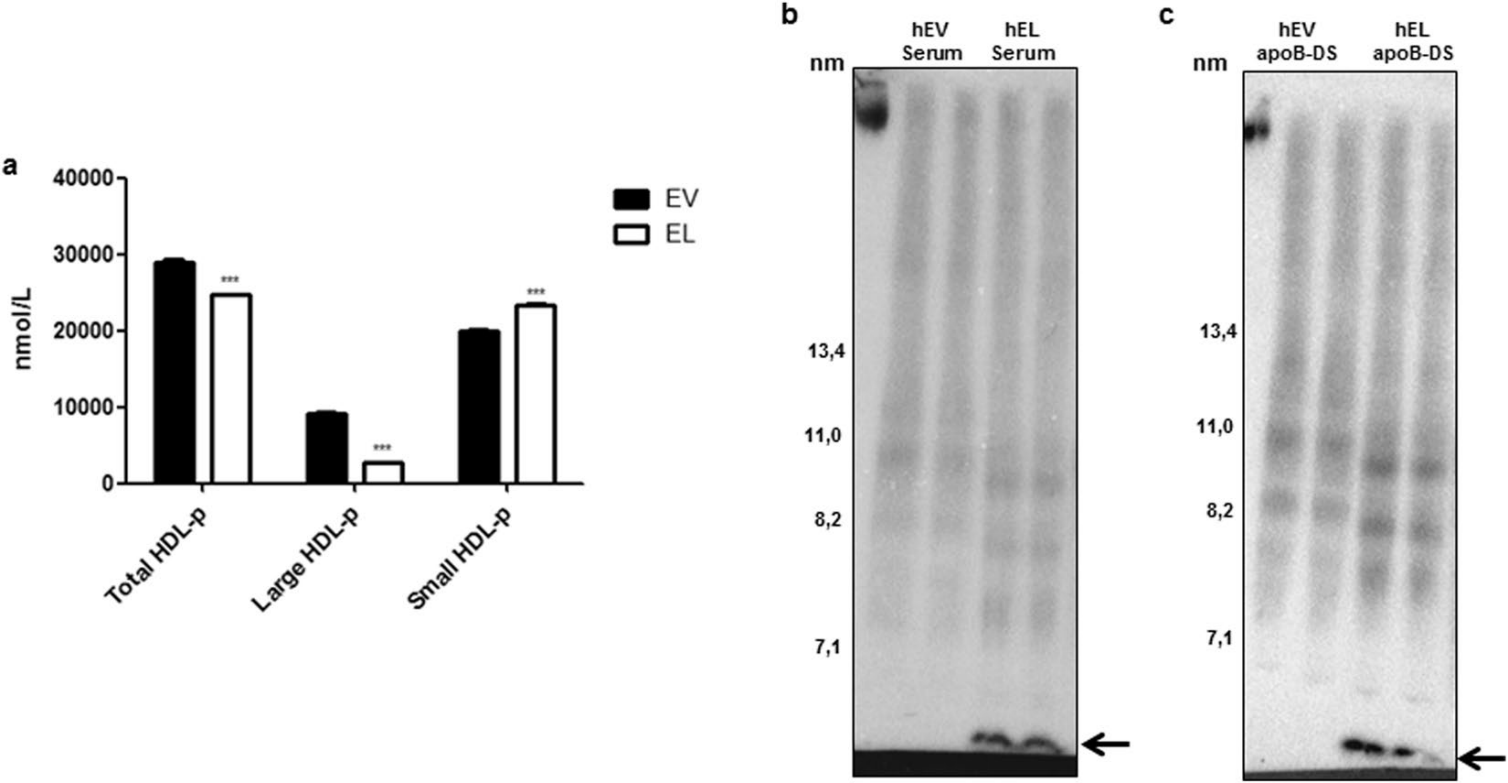
*In vitro* EL-modification of serum decreases HDL size and generates lipid-free/-poor apoA-I (**a**) Concentrations of HDL-particles (HDL-p) in hEV-serum and hEL-serum determined by NMR spectroscopy. Western blotting (apoA-I) following non-denaturing 4–16% polyacrylamide gel electrophoresis of: (**b**) hEV-serum and hEL-serum and (**c**) hEV-apoB-DS and hEL-apoB-DS. Protein size annotations refer to protein marker bands on the membranes. The arrows indicate the position of lipid-free/-poor apoA-I. Results in (**a**) are mean ± SEM of 2 modifications of pool-serum from 8 donors, each measured in duplicates and analysed by unpaired t-test. Results in (**b**) and (**c**) are representative of 5 different modifications of the pool-serum. *P < 0.05, ***P < 0.001.

To provide an explanation for the increased CEC of hEL-serum and the decreased CEC of hEL-HDL, we first examined the impact of EL on HDL size. Nuclear magnetic resonance (NMR) spectroscopy of modified serum revealed that compared to control incubations the EL-modification significantly decreased concentrations of total (p = 0.0127) and large HDL-particles (p = 0.0005) and significantly increased concentrations of small HDL-particles (p = 0.019) compared to EV-controls (Fig. 2a). Furthermore, native gel electrophoresis and subsequent Sudan black staining revealed decreased HDL particle size in both hEL-serum (Supplementary Fig. S2a) and hEL-apoB-DS (Supplementary Fig. S2b) compared to hEV-controls. This was further confirmed by the native gel electrophoresis and subsequent Sudan- and Coomassie- staining as well as apoA-I immunoblotting (Supplementary Fig. S2c,d,e).

Importantly, immunoblotting against apoA-I detected a prominent band smaller than 7.1 nm, in both hEL-serum (Fig. 2b) and hEL-apoB-DS (Fig. 2c), but not in the corresponding hEV-controls. Therefore, EL promotes i*n vitro* the formation of lipid-free/-poor apoA-I, an established mediator of ABCA1-dependent cholesterol efflux.

### Lipid-free/-poor apoA-I is responsible for increased CEC of *in vitro* EL-modified serum

**Figure 3:**
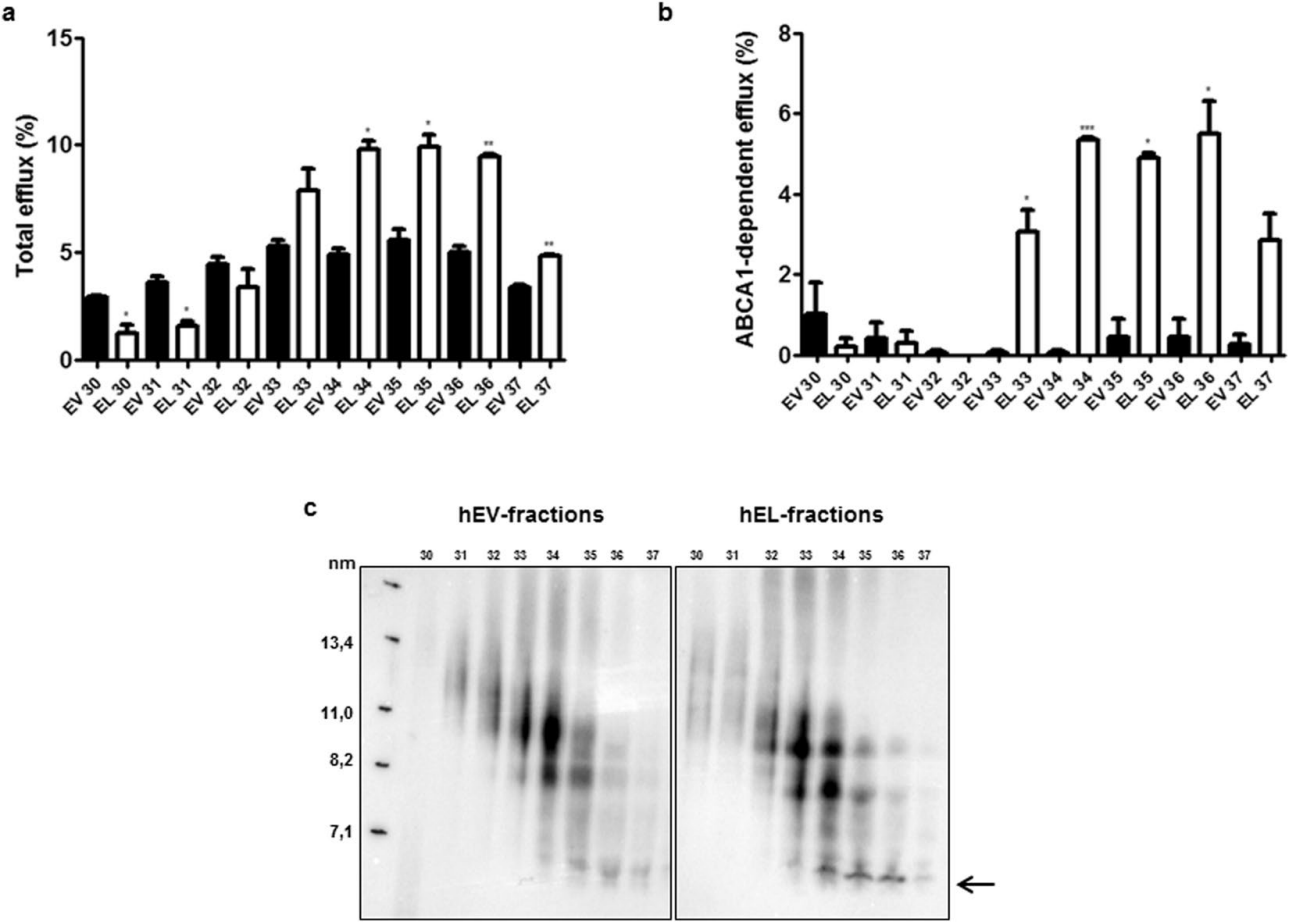
Cholesterol efflux capacity and apoA-I Western blot of the FPLC fractions of hEV-serum and hEL-serum (**a**) Total CEC. (**b**) ABCA1-dependent CEC. (**c**) Western blot (apoA-I) following non-denaturing 4–16% polyacrylamide gel electrophoresis of FPLC fractions of hEV-serum and hEL-serum. Protein size annotations refer to protein marker bands on the membranes. The arrow in (**c**) indicates the position of lipid-free/-poor apoA-I. Results are mean ± SEM of the FPLC fractions of one modification, each measured twice in duplicates and analysed by two-tailed unpaired t-test. *P < 0.05, **P < 0.01, ***P < 0.001.

To substantiate the contribution of lipid-free/-poor apoA-I to the increased CEC of EL-modified serum, we fractionated serum by fast protein liquid chromatography (FLPC) (Supplementary Fig. S3a) and identified apoA-I positive fractions (Supplementary Fig. S3b). As shown in Fig. 3a, the EL-modified serum fractions 30 and 31, containing mature HDL but no lipid-free/-poor apoA-I (Fig. 3c), showed a significantly lower efflux capacity compared to EV-control fractions. The EL fractions 33–37, which contained more lipid-free/-poor apoA-I compared to the corresponding EV fractions (Fig. 3c), exhibited significantly higher total and ABCA1-dependent CEC as compared to EV control fractions (Fig. 3a,b). The ABCA1-independent CEC of the EV and EL fractions was similar (Supplementary Fig. S3c).

### Lipid and apolipoprotein composition of hEL-serum, hEL-apoB-DS and hEL-HDL

**Figure 4:**
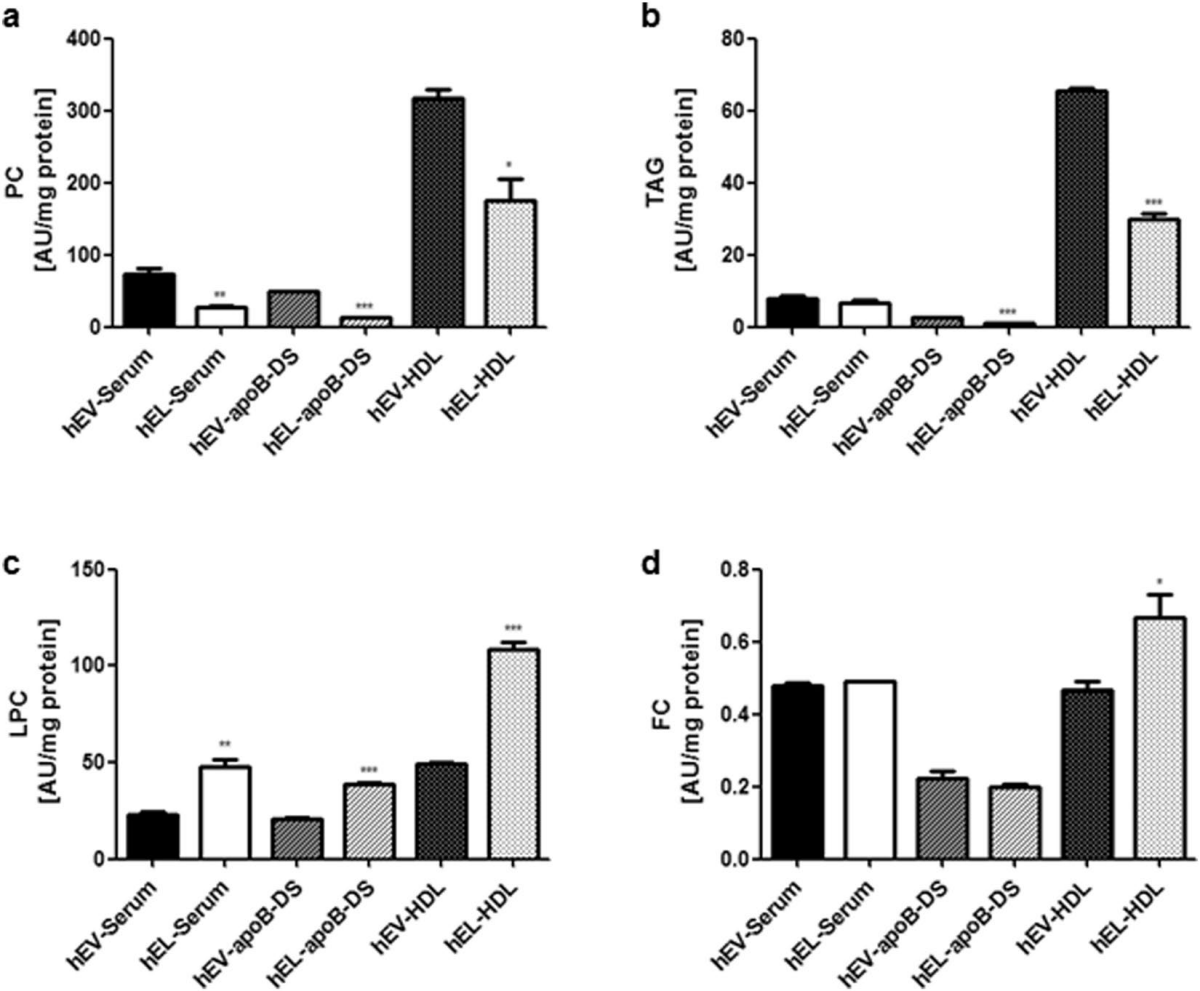
Lipid composition of hEL-serum, hEL-apoB-DS, hEL-HDL and respective hEV-controls Lipids from hEV-serum, hEL-serum, hEV-apoB-DS, hEL-apoB-DS, hEV-HDL and hEL-HDL (corresponding to 300 µg serum or HDL protein) were extracted and (**a**) PC, (**b**) TAG, (**c**) LPC, (**d**) FC were analysed by MS. Results are mean ± SEM of 3 independent modifications of the pool-serum isolated from 8 donors, analysed by two-tailed unpaired t-test. *P < 0.05, **P < 0.01, ***P < 0.001.

By acting on serum *in vitro*, EL promotes the formation of lipid-free/-poor apoA-I, associated with marked changes in serum and HDL functionality. We next assessed to what extent EL alters lipid and apolipoprotein composition of serum and HDL. EL markedly reduced the phosphatidylcholine (PC) (Fig. 4a), phosphatidylethanolamine (PE) (Supplementary Fig. S4a), phosphatidylinositol (PI) (Supplementary Fig. S4b) and triacylglycerol (TAG) content of serum, apoB-DS and HDL, respectively (Fig. 4b). As expected, EL significantly increased the LPC content of serum, apoB-DS and HDL (Fig. 4c). Lysophosphatidylethanolamine (LPE) (Supplementary Fig. S4c) and free cholesterol (FC) (Fig. 4d) were increased only in hEL-HDL, whereas the cholesterol ester (CE) content was unaltered (Supplementary Fig. S4d). Ceramide (Cer) and sphingomyelin (SM) content were only altered in hEL-HDL (Supplementary Fig. S4e,f). While EL did not alter the content of the major HDL apolipoproteins in serum (Supplementary Fig. S4g) and apoB-DS (Supplementary Fig. S4h), the apoA-II content was slightly, but significantly higher in hEL-HDL compared to hEV-HDL (Supplementary Fig. S4i).

### Overexpression of EL in mice decreases HDL abundance and CEC of serum without affecting the size and CEC of isolated HDL

**Figure 5:**
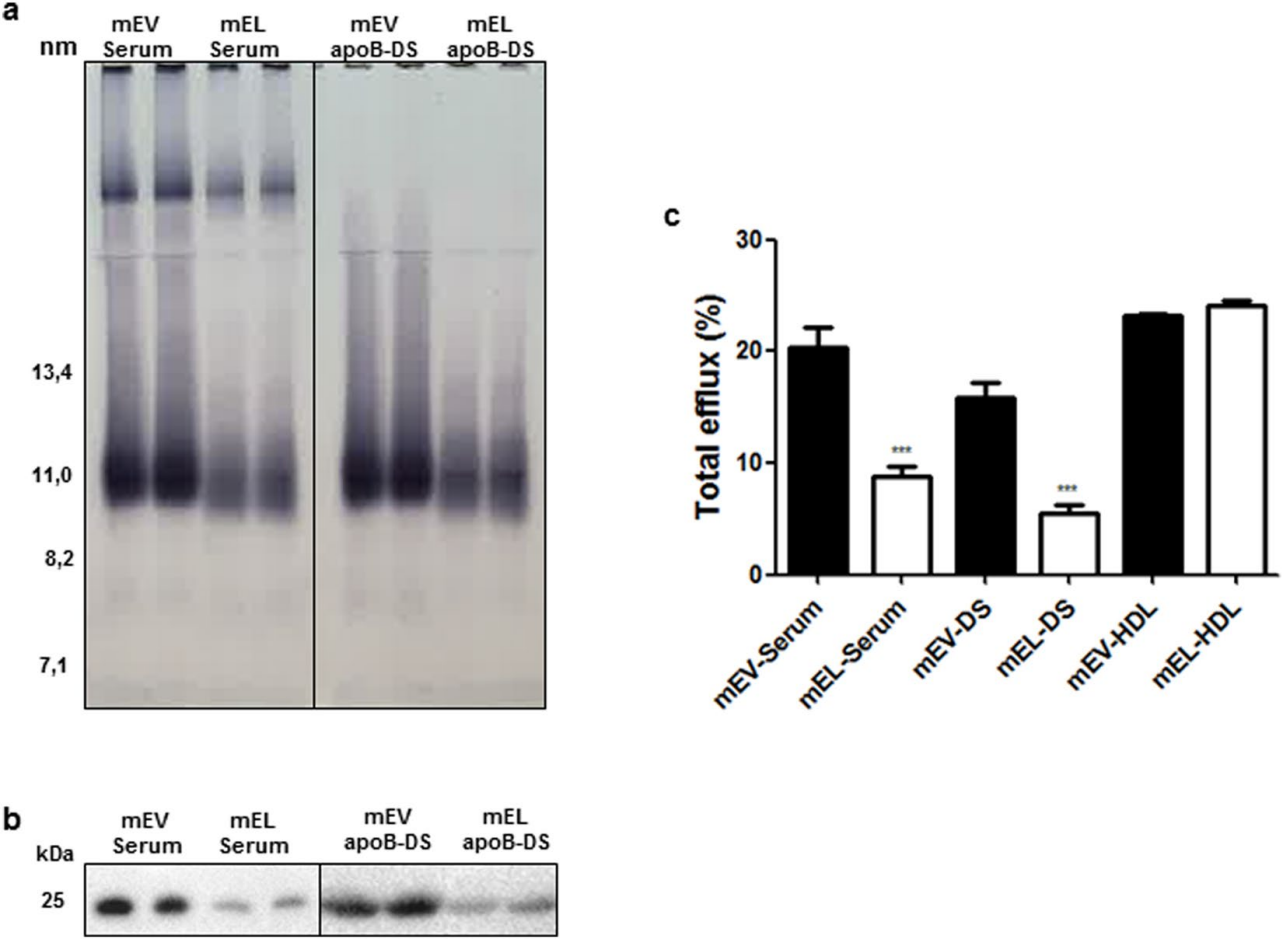
EL overexpression in mice decreases HDL and apoA-I content as well as CEC of serum (**a**) Sudan black staining following non-denaturing 4–16% gel electrophoresis and (**b**) Western blot (apoA-I) following SDS-PAGE of serum and apoB-DS. Full images with 5 lanes per sample are shown in Supplementary Fig. S5a,b. (**c**) Total CEC measured in J774 macrophages following induction of ABCA1 expression. Results are mean ± SEM of 2 independent *in vivo* modifications, with 12 EL-Ad and 4 EV-Ad -transduced mice per each modification, measured twice in duplicates and analysed by two-tailed unpaired t-test. ***P < 0.001.

Prompted by the marked *in vitro* effects of EL on serum and HDL composition and function, we were interested whether EL induces similar changes *in vivo*. For that purpose, human EL was overexpressed in mice using adenoviral transduction. This resulted in a 64% reduction in serum HDL-cholesterol levels compared to EV controls (73 ± 8 mg/dl vs. 26 ± 11 mg/dl, p* = *0.026). Furthermore, Sudan black HDL signal (Fig. 5a) and apoA-I signal obtained by SDS-PAGE and subsequent Western blotting (Fig. 5b and Supplementary Fig. S5a,b) were decreased in mouse serum (mEL-serum) and mouse apoB-DS (mEL-apoB-DS) from EL overexpressing mice. Moreover, EL overexpression altered content of various lipid species in the serum and HDL (Supplementary Fig. S6a–j). In line with the decreased serum HDL content, the total CEC of mEL-serum and mEL-apoB-DS was significantly lower (p* = *0.0002 and p < 0.0001, respectively), as compared to the respective mEV-controls (Fig. 5c). Interestingly, total CEC of mEL-HDL isolated by ultracentrifugation (Fig. 5c) or by FPLC (Supplementary Fig. S7c) was not significantly different from respective mEV-HDL. This was accompanied by unaltered HDL particle size (Supplementary Fig. S8a,b) and significantly altered lipid composition of mEL-HDL as compared to mEV-HDL (Supplementary Fig. S6). From these results we conclude that EL-mediated HDL depletion, but not attenuated CEC of HDL, underlies the decreased CEC of serum from mice which overexpress human EL. Similar results, namely the decreased HDL serum levels and CEC of serum and apoB-DS as well as unaltered CEC and size of isolated HDL, were obtained upon transduction of mice with adenovirus encoding mouse EL (Supplementary Fig. S9a,b,c).

### *In vitro* EL-modification of mouse serum augments CEC and lipid-free/-poor apoA-I content of serum and apoB-DS but decreases CEC of isolated HDL

**Figure 6:**
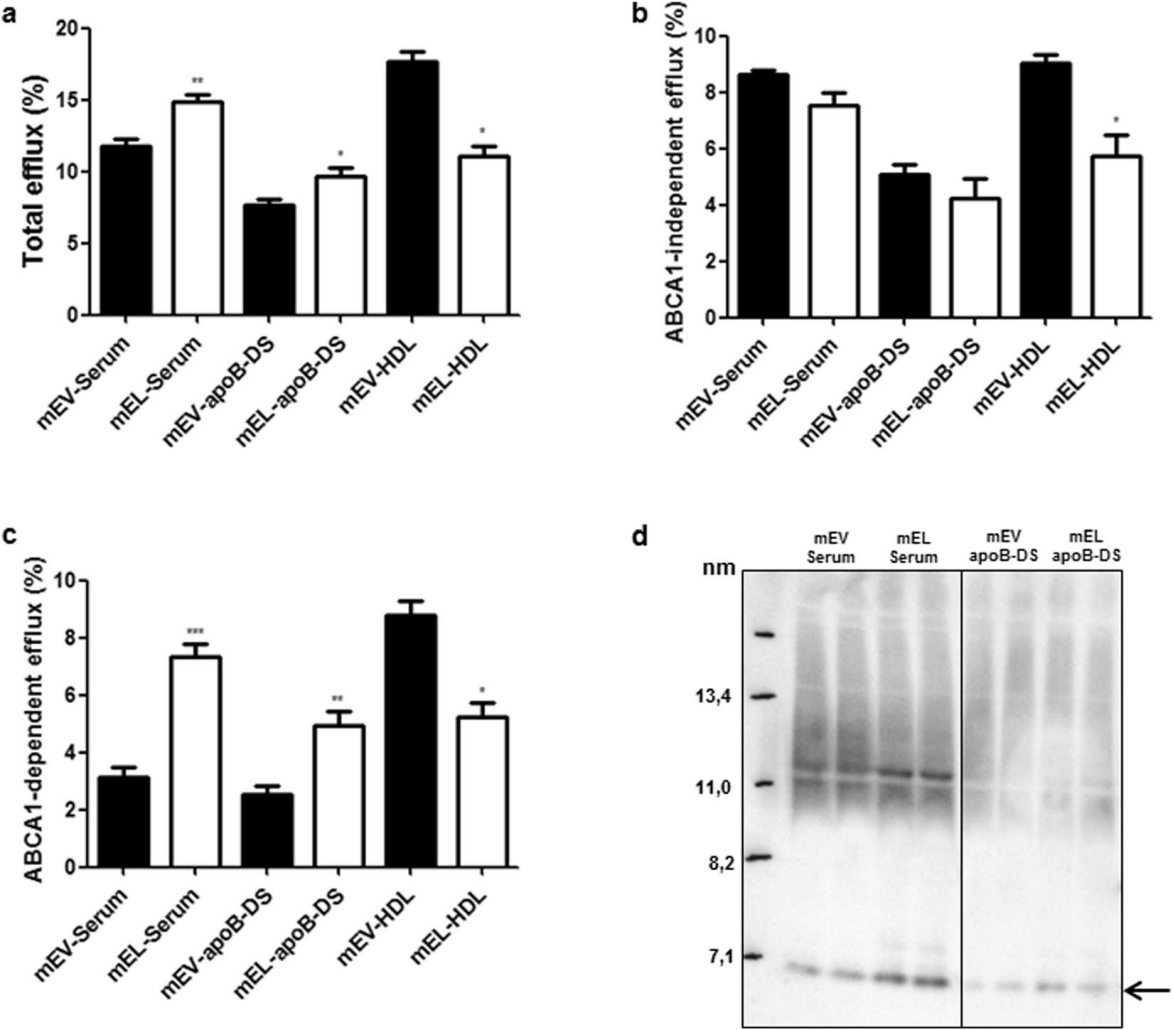
*In vitro* EL-modification of mouse serum augments CEC and lipid-free/-poor apoA-I content of serum and apoB-DS but decreases CEC of HDL Total cholesterol (**a**), ABCA1-independent (**b**) and ABCA1-dependent (**c**) CEC of *in vitro* generated mEL-serum, mEL-apoB-DS, mEL-HDL and respective mEV-controls. d) Western blot (apoA-I) following non-denaturing 4–16% gel electrophoresis. The arrow in (**d**) indicates the position of lipid-free/-poor apoA-I. Results are mean ± SEM of 2 independent modifications of the mouse pool-serum, each measured twice in duplicates and analysed by two-tailed unpaired t-test. *P < 0.05, **P < 0.01, ***P < 0.001.

To examine whether the conflicting data regarding effects of EL on CEC obtained *in vitro* and *in vivo* simply reflect the differences in species-specific features of human and mouse serum, or in features of experimental models (closed *in vitro* vs. open *in vivo*), we modified mouse serum with human EL *in vitro*. We found that EL augmented total and ABCA1-dependent CEC of mouse serum (Fig. 6a,c), accompanied by increased serum content of lipid-free/-poor apoA-I (Fig. 6d). EL decreased CEC of mouse HDL (Fig. 6a,b,c) without altering HDL size (Supplementary Fig. S10).

## Discussion

In the present study, we examined in detail the impact of EL on composition and function of serum and isolated HDL *in vitro* and *in vivo*. We provide evidence that EL induces complex modifications in serum, associated with marked alterations in HDL structure, composition, function and metabolism.

We found that *in vitro* modification of both human and mouse serum with EL augments CEC of serum, but decreases CEC of HDL isolated from EL-modified serum. Most recent data showed that small, dense HDL subfractions and lipid-free/-poor apoA-I are the most efficient mediators of ABCA1-mediated cholesterol efflux^17,18^. In good agreement with these reports we observed in the present study that the generation of lipid-free/-poor apoA-I, underlies the augmentation of CEC of *in vitro* EL-modified serum. In contrast, EL induced formation of intermediate sized phospholipid depleted HDL particles (8–8.2 nm) exhibited significantly decreased CEC likely due to a decreased affinity of EL-HDL to scavenger receptor class B1^6^. Moreover, it is well established that HDL associated FC, SM and Cer reduce fluidity of surface phospholipids and in turn impair CEC^18–20^. Interestingly, FC was increased in the *in vitro* generated EL-HDL (Fig. 4d and Supplementary Fig. S11h), possibly contributing to the decreased CEC of the *in vitro* generated EL-HDL. Induction of cholesterol efflux from HepG2 cells by EL-derived LPC^21^ during *in vitro* HDL modification may be responsible for the increased FC content observed in the *in vitro* generated EL-HDL.

Since HDL from *in vitro* EL-modification of human and mouse serum exhibited similar degree of CEC- reduction (Fig. 1 and Fig. 6), accompanied by similar degree of PE- and PI-, but not PC- reduction (Fig. 4a, Supplementary Fig. S4a,b and Supplementary Fig. S11a,b,c), our results fit in with the results of previous studies demonstrating that the efficiency of cholesterol efflux is correlated with HDL-phospholipid content^15,22–24^, and additionally argue for the role of minor phospholipid species, PE and PI, in CEC of HDL. The abundance of another minor phospholipid species, phosphatidylserine, which is an established enhancer of HDL functionality, including CEC^25^, could not be determined due to technical limitation. In contrast to decreased CEC of *in vitro* generated EL-HDL, the CEC of *in vivo* generated EL-HDL was unaltered, likely due to the absence of phospholipid reduction and FC enrichment (Supplementary Fig. S6a,b,c,h), the lipid composition alterations found only in *in vitro* generated EL-HDL.

An unexpected finding of the present study was increased PE in the *in vivo* generated mEL-serum and mEL-HDL (Supplementary Fig. S6b) as well as in the *in vitro* generated mEL-serum (Supplementary Fig. S11b). This contrasts to PE being an established substrate for EL^16^ as well as to markedly decreased PE plasma levels in EL overexpressing mice^12^. It remains to be determined whether the rate and duration of EL overexpression or components of mouse serum underlie the conflicting results.

In sharp contrast to a markedly increased CEC of the *in vitro* EL-modified human serum accompanied by accumulation of lipid-free/-poor apoA-I, the CEC of the *in vivo* EL-modified serum was profoundly decreased and accompanied by markedly decreased HDL serum levels (Fig. 5a,b). Importantly, in line with the notion, that lipid-free/-poor apoA-I is rapidly cleared via kidney in mice^26,27^, we observed no accumulation of lipid-free/-poor apoA-I in the serum from EL-overexpressing mice (Supplementary Fig. S12). Together, our data suggest that both the enhanced EL-mediated HDL catabolism and rapid clearance of lipid-free/-poor apoA-I underlie the decreased CEC of serum from EL-overexpressing mice.

Interestingly, *in vitro* EL-modification of human serum resulted in a decreased HDL particle size (Supplementary Fig. S2), whereas neither *in vitro* nor *in vivo* EL-modification of mouse serum affected HDL particle size (Supplementary Figs S8 and S10). This difference between human and mouse HDL might be explained by a more pronounced decrease in PC and TAG by EL-associated phospholipase and TAG-lipase activity^28^ in hEL-HDL (Fig. 4a,b) than in mEL-HDL (Supplementary Figs S6a,f and S11a,f). Considering the important role of hepatic lipase (HL) in HDL metabolism^29^ the increased TAG content in HDL of EL overexpressing mice (Supplementary Fig. S6f) might reflect a decreased cleavage of HDL-associated TAG by HL. However, we observed no change in hepatic LipC (HL) mRNA levels upon adenoviral EL-overexpression in mice (Supplementary Fig. S13). Therefore, we assume that it is unlikely that an altered HL activity causes the increased TAG content of HDL in EL-overexpressing mice. The possibility that unaltered size of mEL-HDL reflects the inefficient cleavage of mouse HDL by human EL was excluded by *in vivo* overexpression of mouse EL, which also failed to decrease the size of mouse HDL (Supplementary Fig. S9).

Decreased CEC of the serum from EL-overexpressing mice observed in our study is consistent with decreased CEC of serum from mice in which EL overexpression was a consequence of profurin-overexpression^16^. However, another study reported increased CEC of serum from EL-overexpressing human apoA-I transgenic mice despite a massive reduction in apoA-I serum levels^15^. A possible explanation for these discrepant findings could be the slower catabolic rate of human, compared to mouse lipid- free/-poor apo A-I in the EL-overexpressing mice and consequently the existence of a pool of lipid- free/-poor human apoA-I in mice, capable of promoting cholesterol efflux.

Attenuation of serum CEC by EL-overexpression in mice observed in the present and a previous study^16^, together with an enhanced CEC of serum from EL deficient mice^30,31^ as well as from subjects with loss of function mutations in a gene encoding EL^5^, clearly argues for EL being an attenuator of serum CEC. Interestingly, however, in the present study, the EL action on human and mouse serum *in vitro* increased CEC of serum (but not of isolated HDL) due to generation and accumulation of lipid-free/-poor apoA-I. Therefore, EL appears to have the ability to generate potent cholesterol acceptors by acting on serum. It remains to be determined whether those cholesterol acceptors are rapidly catabolized as observed in mice or accumulate in human plasma during EL-upregulation.

Based on our results we conclude that EL-mediated generation and accumulation of lipid-free/-poor apoA-I underlies increased CEC of the *in vitro* generated EL-serum and EL-apoB-DS. Quite the opposite, EL overexpression in mice depletes HDL without increasing lipid-free/-poor apoA-I thereby profoundly impairing CEC of serum. Further investigations are required to determine whether EL upregulation in humans increases lipid-free/-poor apoA-I and in turn CEC of human serum *in vivo*.

## Methods

### Cell culture

HepG2 cells (ATCC^®^, HB-8065^TM^)^32^ were maintained in DMEM supplemented with 2 mM glutamine, 1% PS (100 U/mL penicillin, 100 µg/mL streptomycin) and 10% fetal calf serum (FCS). J774.2 macrophages (Sigma-Aldrich, Vienna, Austria; #85011428)^33^ were maintained in RPMI1640 medium with 10% FCS.

### Preparation of heparin media

Heparin media were prepared as described in our previous reports^10,11^.

### Preparation of human and mouse *in vitro* EL-modified and EV-control serum

Human serum was obtained after overnight fasting from 8 healthy volunteers (4 females and 4 males). The local Ethics Committee of the Medical University of Graz approved all experimental protocols related to human volunteers (28-186 ex 15/16). Written informed consent was obtained from each subject in compliance with Good Clinical Practice. To obtain mouse serum blood was collected from the right ventricle from non-fasted mice anesthetized with sevorane (AbbVie, Vienna, Austria). All experimental protocols related to animal experiments were approved by the Austrian Federal Ministry for Science and Research (BMWF-66.010/0133-II/3b/2012). All experiments were performed in accordance with relevant guidelines and regulations. To obtain serum, blood was incubated for 30 min at room temperature (RT) followed by centrifugation (3000 x g) at 4 °C for 15 min. Serum was stored at −80 °C or used immediately for modification. HepG2 cells (2 × 10^6^) were plated onto 60 mm dishes and incubated under standard conditions as described above. After 24 h, cells were washed once with DMEM without FCS (pH 7.4) and transduced with multiplicity of infection of 20 using recombinant adenoviruses encoding human EL (hEL-Ad) or empty adenovirus containing no recombinant cDNA (EV-Ad)^11^ in DMEM without FCS for 2 h. After removal of infection media cells were incubated with fresh DMEM containing 10% fetal calf serum (FCS) for 20 h. Thereafter, cells were washed once with DMEM without FCS and each plate was incubated under cell culture conditions with 1.8 mL of 50% pooled human or mouse serum in DMEM without FCS, for 8 h. After incubation, the serum was collected and spun at 1100 × g for 3 min to remove cellular debris. *In vitro* generated hEL-serum, hEV-serum, mEL-serum and mEV-serum were used for the preparation of respective apoB-DS as described below.

### Preparation of *in vivo* EL-modified serum and control EV serum

Male C57BL/6 mice (9-12 weeks old) were injected with 3.2 × 10^8^ plaque forming units (p.f.u.) of EV-Ad, human EL-Ad or mouse EL-Ad^10,11^ in 100 µL of PBS via the tail vein. Blood was collected from the right ventricle 48 h after virus injection from non-fasted mice. Serum was stored at −80 °C or used immediately for further experiments or preparation of apoB-DS as described below. During injection into the tail vein as well as during bleeding by right ventricle puncture, the mice were anesthetized by sevorane (AbbVie, Vienna, Austria).

### ApoB-depletion of serum

ApoB-DS was prepared by the addition of 40 μL polyethylene glycol (20% in 200 mmol/L glycine buffer) to 100 μL mouse serum or 100 μL 50% (diluted in DMEM without FCS: v/v) human serum. The samples were incubated at room temperature for 20 minutes and the supernatant was recovered after centrifugation (10.000 rpm, 30 minutes, 4 °C) as described^34^.

### Isolation of HDL from modified serum

HDL was isolated by a one-step density gradient ultracentrifugation method using long centrifuge tubes (16 × 76 mm; Beckman), as described^34^. Briefly, the density-adjusted serum (1.24 g/mL with potassium bromide) was layered underneath a potassium bromide-density solution (1.063 g/mL). Samples were centrifuged at 330.000 x g for 6 h (centrifuge: Beckman Optima L-80 ultracentrifuge, rotor: Sorvall T-1270). Thereafter, the collected HDL was concentrated by Viva Spin Tubes (Sartorius, Vienna, Austria), desalted by gel filtration on Sephadex PD-10 columns (GE Healthcare, Munich, Germany) and either used directly or stored at −80 °C for further experiments.

### FPLC of *in vitro* and *in vivo* modified serum

A pool of 200 µL of each modification was subjected to FPLC on a Pharmacia FPLC system (Pfizer Pharma, Karlsruhe, Germany) equipped with a Supherose 6 column (Amersham Biosciences, Piscataway, NJ). Lipoproteins were eluted with 10 mmol/L Tris-HCl, 1 mmol/L EDTA, 0.9% NaCl, and 0.02% NaN_3_ (pH 7.4). The total cholesterol (TC) (Greiner Diagnostics AG, Bahlingen, Germany) concentrations in 0.5 mL fractions were determined spectrophotometrically.

### Analysis of modified serum by NMR spectroscopy

hEL-serum and hEV-serum were generated as described above with exception that 100% human serum was incubated with EL-overexpressing or EV-control HepG2 cells for 8 h. EL-modified and EV-control samples were analysed using the *AXINON* ^*®*^ *lipoFIT* ^*®*^ *–S100* test system (Numares Health, Regensburg, Germany) as described in our previous reports^35,36^.

### Measurements of CEC

J774 macrophages plated on 48-well plates (300.000 cells/well) were labeled with 1 μCi/mL [^3^H]-cholesterol (Perkin Elmer, Boston, MA, USA) for 24 hours. To upregulate ABCA1, the cells were stimulated with serum-free DMEM containing 0.3 mmol/L 8-(4-chlorophenylthio)-cyclic AMP (Sigma, Darmstadt, Germany) for 6 hours. After labeling, the cells were washed and the [^3^H]-cholesterol efflux was determined by incubating the cells with 2.8% of human or mouse serum, apoB-DS or with human or mouse HDL (2 µg HDL protein) or FPLC fractions (30%; v/v) for 4 hours. The cholesterol efflux was expressed as the radioactivity in the medium relative to total radioactivity in the medium and cells. ABCA1- dependent cholesterol efflux was calculated by subtracting ABCA1-independent efflux (obtained in J774 macrophages without ABCA1 upregulation) from the total efflux (measured in cells with upregulated ABCA1). All steps were performed in the presence of 2 μg/mL of the acyl coenzyme A cholesterol acyltransferase inhibitor Sandoz 58-035 (Sigma, Darmstadt, Germany).

### Determination of apolipoprotein composition of serum, apoB-DS and HDL

Apolipoproteins were determined by immunoturbidimetry using reagents from DiaSys (Holzheim, Germany) and standards from Siemens (Marburg, Germany; apoAI, apoB, apoE) and Kamiya Biomedical (Seattle, WA, USA; apoAII, apoCII, apoCIII). All measurements were performed on an Olympus AU640 automatic analyser. The coefficients of variation (between day) were <5%.

### Non-denaturing gradient-gel electrophoresis and Western Blotting

Aliquots of serum (1.5 µL), apoB-DS (2 µL), HDL (10 µg) and FPLC fractions (15 µL) were electrophoresed on 4–16% non-denaturing polyacrylamide gels upon dilution with native sample buffer (LifeTechnologies, Vienna, Austria). Electrophoresis was done in a running buffer (Invitrogen, Vienna, Austria) at 125 V for 4 h at room temperature. Gels were stained with Sudan black (Sigma-Aldrich, Vienna, Austria) or were fixed with 10% sulfosalicylic acid for 30 min and then stained with Coomassie Brillant Blue G250 or used for Western blot analysis. The high molecular weight marker NativeMark (LifeTechnologies, Vienna, Austria) was used as standard. Separated proteins were transferred to polyvinylidene difluoride (PVDF) membrane (Carl Roth, Karlsruhe, Germany) with blotting buffer (Tris, Glycine, EDTA, sodium azide) at 400 mA at 4 °C for 75 min. Membranes were blocked at RT in 10% skim milk for 1 h followed by overnight incubation at 4 °C with apoA-I antibody obtained from Novus biological (NB100-65491, LOT 120810, dilution 1:3000, Littleton, CO, USA) for detection of human apoA-I or with *in house* generated apoA-I antibody^30^ (dilution 1:1000) kindly provided by Dr. G.M. Kostner, for detection of mouse apoA-I. After washing and incubation with appropriate secondary antibody (Dako, Vienna, Austria) protein signals were visualized by incubation with Millipore Western Blotting Substrate (Millipore Corporation, Billerica, USA) using ChemiDoc system (Bio-Rad Laboratories, Vienna, Austria).

### SDS-PAGE and Western blotting

For the analyses of EL in heparin media 40 µL of heparin media were supplemented with 6 x loading buffer [20% (w/v) glycerol, 5% (w/v) SDS, 0.15% (w/v) bromophenol blue, 63 mmol/L Tris-HCl, pH6.8 and 5% (v/v) ß-mercaptoethanol], and boiled for 10 min before loading. Samples were analyzed by SDS-PAGE (10% gel) and with subsequent immunoblotting using EL-specific antibody exactly as described^10^. Mouse apoA-I content was analyzed in aliquots of serum (1.5 µL), apoB-DS (2 µL) and FPLC fractions (10 µL). Samples were supplemented with 6 x loading buffer, boiled for 10 min and electrophoresed on 12% SDS-PAGE. After blotting the membranes were incubated at 4 °C overnight with mouse anti-apoA-I antibody (Santa Cruz Biotechnology, sc-30089, LOT D2913, dilution 1:500, Heidelberg, Germany). Following washing and incubation with appropriate secondary antibody (Dako, Vienna, Austria), protein signals were visualized by incubation with Millipore Western Blotting Substrate (Millipore Corporation, Billerica, USA) using ChemiDoc system (Bio-Rad Laboratories, Vienna, Austria).

### Targeted lipidomic analysis

Total lipids of *in vivo* and *in vitro* experiments (300 µg protein) were extracted twice according to Folch *et al*.^37^ using chloroform/methanol/water (2/1/0.6, v/v/v) containing 500 pmol butylated hydroxytoluene, 1% acetic acid, and 100 pmol of internal standards (ISTD, 17:0-17:0 PC, 19:0-19:0 PC, 17:0-17:0 PE, 17:0 FA, d18:1/17:0 Cer, 14:0-14:0 DG, 17:0 LPC, 17:0-17:0-17:0 TG, 15:0-15:0-15:0 TG, Avanti Polar Lipids) per sample. Extraction was performed under constant shaking for 60 min at room temperature (RT). After centrifugation at 1,000 × *g* for 15 min at RT the lower organic phase was collected. 2.5 mL chloroform was added to the remaining aqueous phase and the second extraction was performed as described above. Combined organic phases of the double-extraction were dried under a stream of nitrogen and resolved in 200 µL methanol/2-propanol/water (6/3/1, v/v/v) for UPLC-TQ analysis. Chromatographic separation was modified after Knittelfelder *et al*.^38^ using an AQUITY-UPLC system (Waters Corporation), equipped with a Kinetex EVOC18 column (2.1 × 50 mm, 1.7 µm; Phenomenex) starting a 25 min gradient with 100% solvent A (MeOH/H2O, 1/1, v/v; 10 mM ammonium acetate, 0,1% formic acid). A EVOQ Elite™ triple quadrupole mass spectrometer (Bruker) equipped with an ESI source was used for detection. Lipid species were analyzed by selected reaction monitoring (PC: [MH] + to m/z 184, 25 eV, PE: [MH] + to −m/z 141, 20 eV, PI: [M-H]- to corresponding [FA]−, 50 eV, LPC: [MH] + to m/z 184, 22 eV, LPE: [MH] + to −m/z 141, 17 eV, Cer: [MH] + to m/z 264, 22 eV, TG: [MNH4] + to corresponding [DG-H2O]+, 22 eV, DG: [MNH4] + to [RCOO + 58] + , 15 eV, CE: [MNH4] + to m/z 369, FC: [M-H2O] + , 0 eV, FA: [M-H]-, 0 eV, SM: [MH] + to m/z 184, 23 eV). Data acquisition was done by MS Workstation (Bruker). Data were normalized for recovery and extraction- and ionization efficacy by calculating analyte/ISTD ratios.

### RNA isolation and quantitative real-time PCR analysis

Total RNA from 50 mg liver was isolated using TriFast™ reagent according to the manufacturer’s protocol (Peqlab, Erlangen, Germany). Two μg of total RNA was reverse transcribed using the High Capacity cDNA Reverse Transcription Kit (Applied Biosystems, Carlsbad, CA). Quantitative real-time PCR was performed on a Roche LightCycler 480 (Roche Diagnostics, Palo Alto, CA) using the GoTaq^®^ qPCR MasterMix (Promega, Madison, WI). Samples were analyzed in duplicate and normalized to the expression of cyclophilin A as reference gene. Expression profiles and associated statistical parameters were determined using the 2^−*ΔΔCT*^ method. Primers for mouse cyclophilin A (fw: CCATCCAGCCATTCAGTCTT; rev: TTCCAGGATTCATGTGCCAG), and mouse LipC (fw: CCATCCAGCCATTCAGTCTT; rev: TTCCAGGATTCATGTGCCAG) were from Life Technologies (Vienna, Austria) and for human LipG we used Primer Assay QT00078967, purchased from Qiagen (Hilden, Germany).

### Statistical analyses

Data are represented as the means ± standard error of mean (S.E.M.). Differences between EV- and EL- samples were assessed by two-tailed unpaired *t*-test using Graph Pad Prism 5.0. Statistically significant differences between groups are indicated by *P*-values of <0.05 (*), <0.01 (**), or <0.001 (***).

### Data Availability

All data generated or analysed during this study are included in this published article (and its Supplementary Information files).

## Electronic supplementary material

Supplementary Figures
