# Supplementary material for: Impact of Endothelial Lipase on Cholesterol Efflux Capacity of Serum and High-density Lipoprotein
Source: Sci Rep. 2017 Oct 2;7:12485. doi: 10.1038/s41598-017-12882-7 (PMC5624901; doi:10.1038/s41598-017-12882-7)
Supplement: Supplementary file 1 — Supplementary Figures [file 41598_2017_12882_MOESM1_ESM.pdf]

Impact of Endothelial Lipase on Cholesterol Efflux Capacity of Serum and High-density Lipoprotein

Irene Schilcher, Sabine Kern, Andelko Hrzenjak, Thomas O. Eichmann, Tatjana Stojakovic, Hubert Scharnagl, Madalina Duta-Mare, Dagmar Kratky, Gunther Marsche and Saša Frank

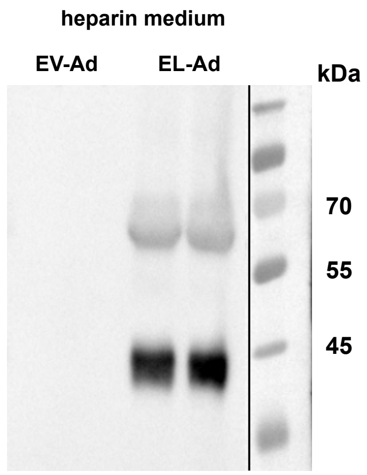

Supplementary Fig. S1. Overexpression of human EL in HepG2 cells
Western blot analysis of EL in heparin supplemented media of HepG2 cells transduced with EV-Adenovirus (EV-Ad) or EL-Ad.

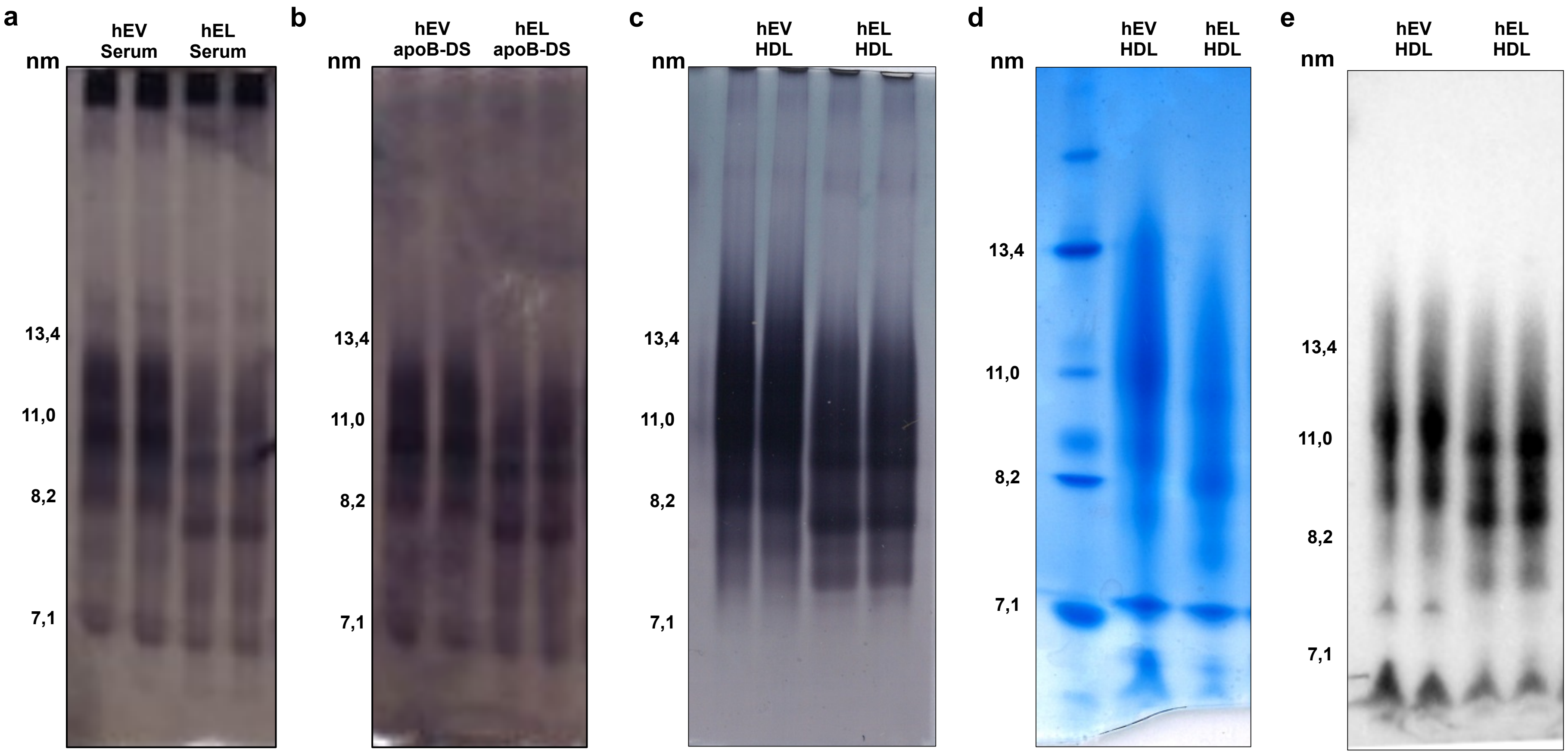

Supplementary Fig. S2. HDL size is decreased upon EL modification of human serum *in vitro*

Aliquots of human (h) EV-serum or hEL-serum (1.5 μL), hEV-apoB-DS or hEL-apoB-DS (2 μL) and hEV-HDL or hEL-HDL (10 μg protein) were electrophoresed on 4-16% non-denaturing polyacrylamide gels followed by Sudan black (a, b, c), Coomassie staining (d) or apoA-I Western blotting (e). Protein size annotations refer to protein marker bands on the membranes. Results are representative of 5 different modifications of the pool-serum isolated from 8 donors.

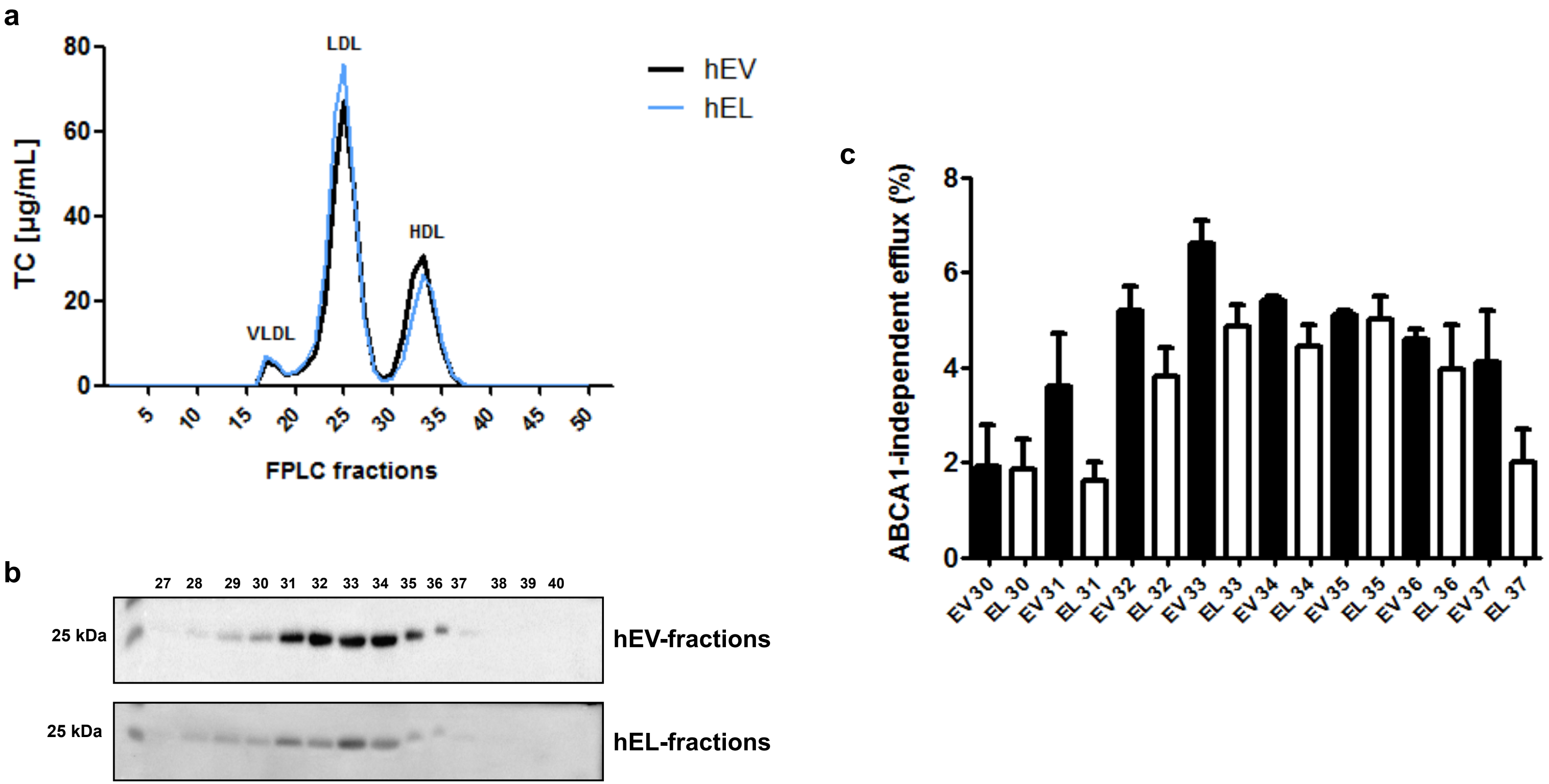

Supplementary Fig. S3. Western blot and CEC of the FPLC fractions of hEV-serum and hEL-serum

a) FPLC fractions 27 – 37 were analysed by b) SDS-PAGE and apoA-I Western blot. c) ABCA1-independent CEC was measured in J774 macrophages under basal conditions. Protein size annotations refer to protein marker bands on the membranes. Shown is a representative experiment out of 2 independent modifications, both giving similar results. Results are mean ± SEM of the FPLC fractions of one modification, each measured twice in duplicates and analysed by unpaired t-test.

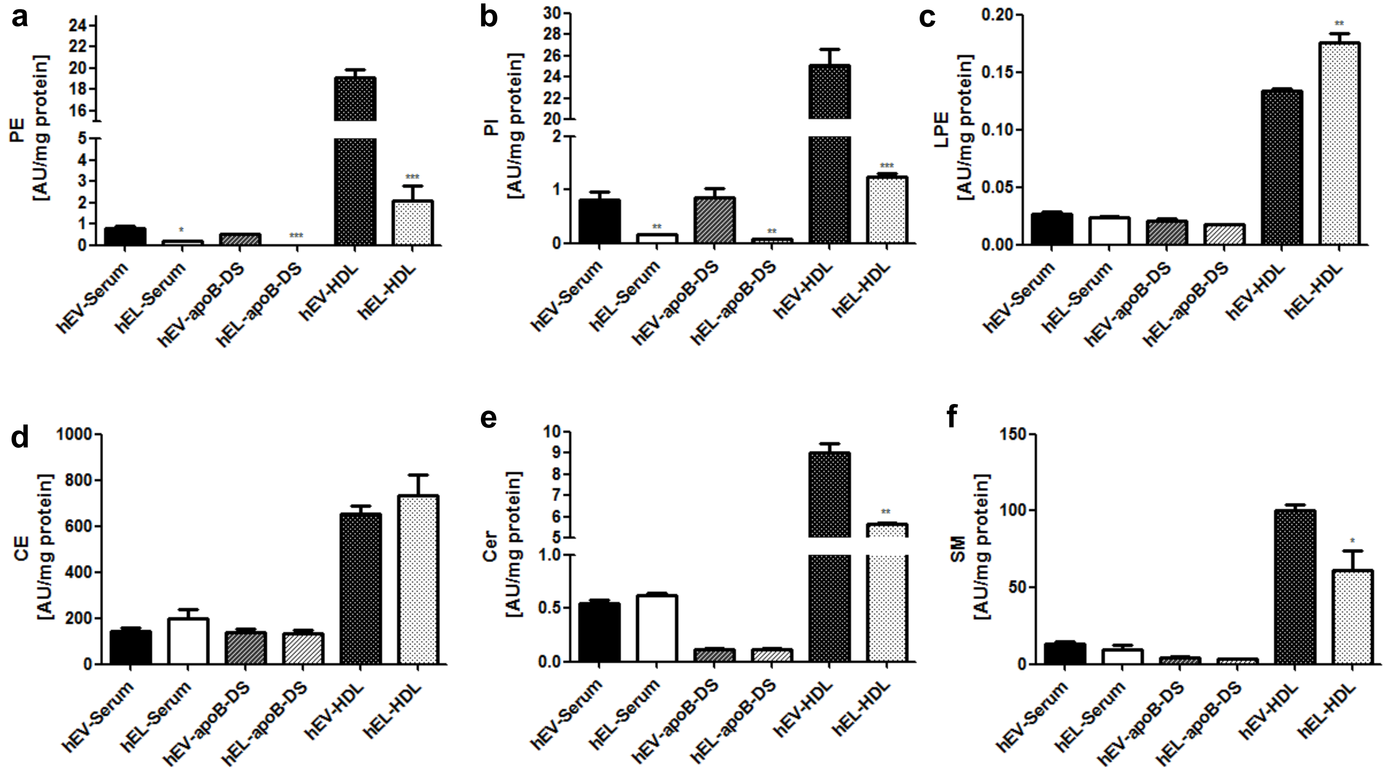

Supplementary Fig. S4 (a-f). Lipid and apolipoprotein composition of hEL-serum, hEL-apoB-DS, hEL-HDL and respective hEV-controls

Lipids from hEV-serum, hEL-serum, hEV-apoB-DS, hEL-apoB-DS, hEV-HDL and hEL-HDL (corresponding to 300 μg serum or HDL protein) were extracted and (a) PE, (b) PI, (c) LPE, (d) CE, (e) Cer, (f) SM were analysed by MS. Results are mean ± SEM of 3 independent modifications of the pool-serum isolated from 8 donors. $^{*}P < 0.05$, $^{**}P < 0.01$, $^{***}P < 0.001$.

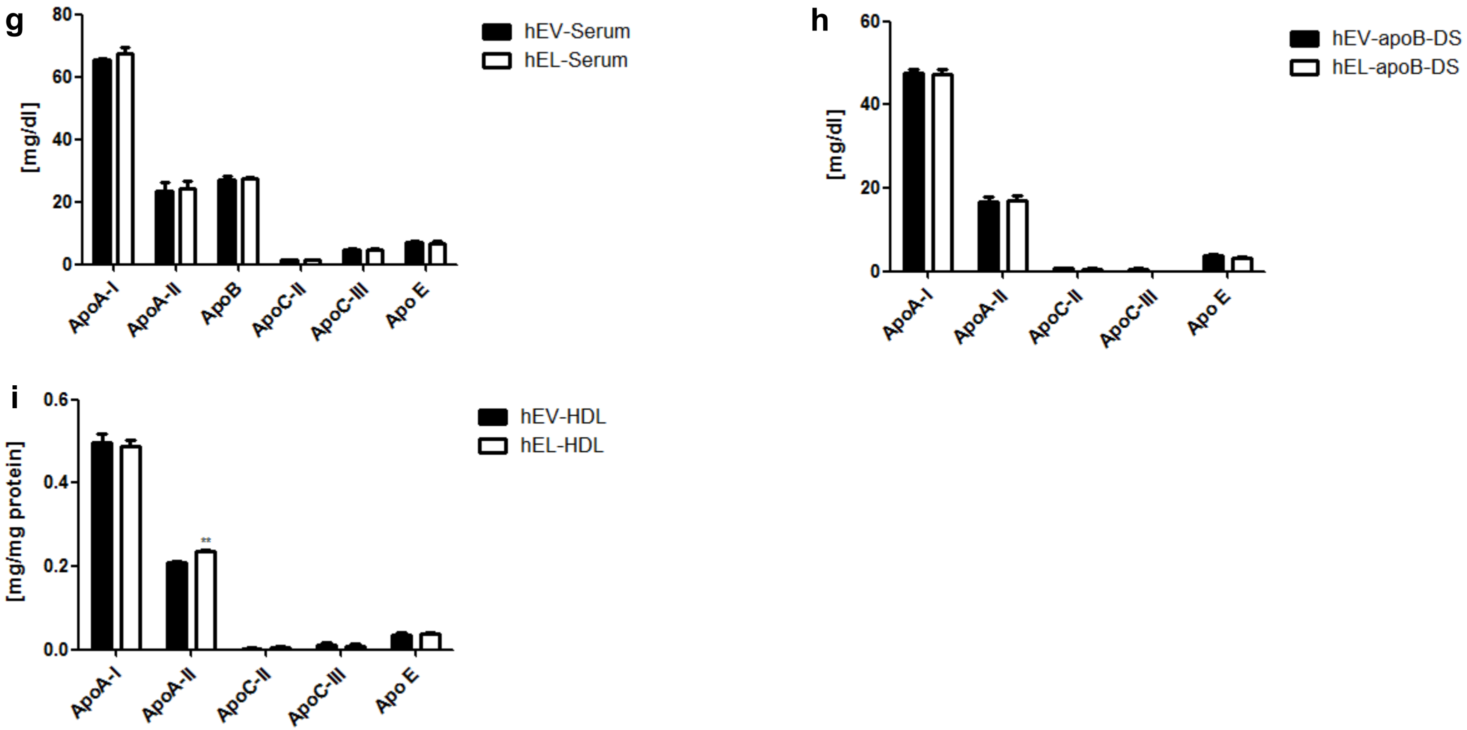

Supplementary Fig. S4 (g-i). Lipid and apolipoprotein composition of hEL-serum, hEL-apoB-DS, hEL-HDL and respective hEV-controls

Apolipoprotein composition of serum (g), apoB-DS (h) and HDL (i) was analysed by immunoturbidimetry. Results are mean ± SEM of 3 independent modifications of the pool-serum isolated from 8 donors. $^*P < 0.05$, $^{**}P < 0.01$.

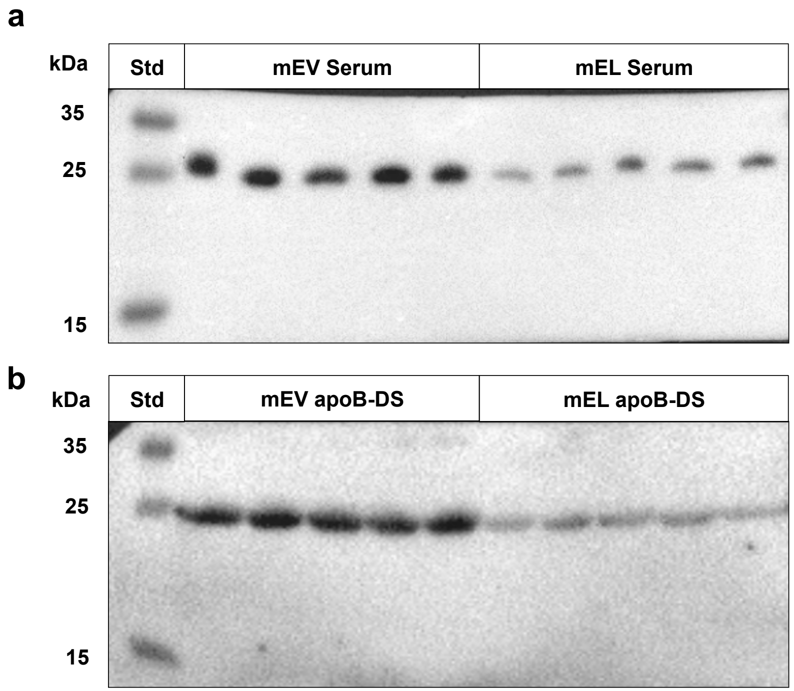

Supplementary Fig. S5: EL overexpression in mice decreases HDL and apoA-I content as well as CEC of serum
Representative Western blot (apoA-I) following SDS-PAGE of (a) serum and (b) apoB-DS of 2 independent *in vivo* modifications, with 12 EL-Ad and 4 EV-Ad -transduced mice per each modification.

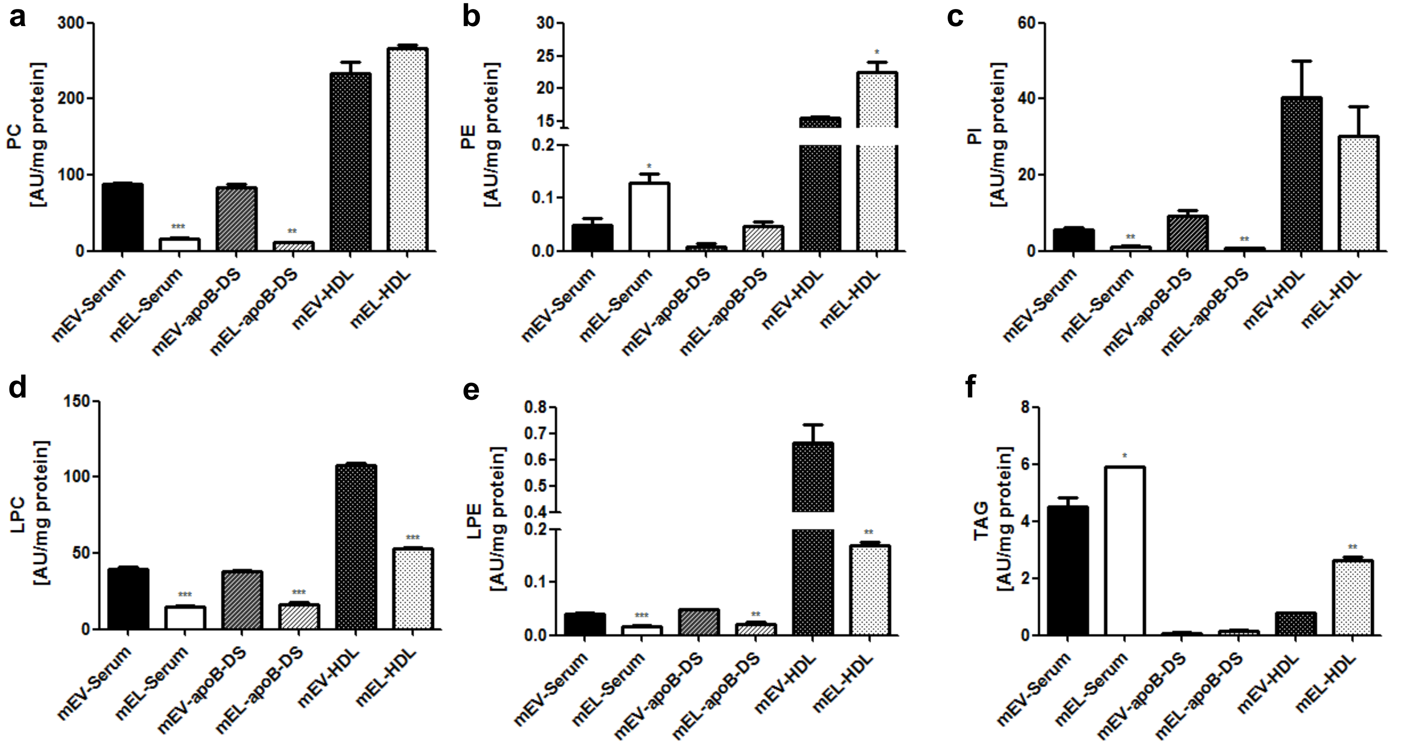

Supplementary Fig. S6 (a-f). EL overexpression in mice alters lipid composition of serum, apoB-DS and HDL
Lipids from mouse (m) EV-serum, mEL-serum, mEV-apoB-DS, mEL-apoB-DS, mEV-HDL and mEL-HDL (corresponding to 300 μg serum or HDL protein) were extracted and (a) PC, (b) PE, (c) PI, (d) LPC, (e) LPE, (f) TAG were analysed by MS. Results are mean ± SEM of 3 measurements for each serum, apoB-DS and HDL obtained in 2 independent *in vivo* modifications, with 12 EL-Ad- and 4 EV-Ad-transduced mice per each modification. *$P < 0.05$, **$P < 0.01$, **$P < 0.001$.

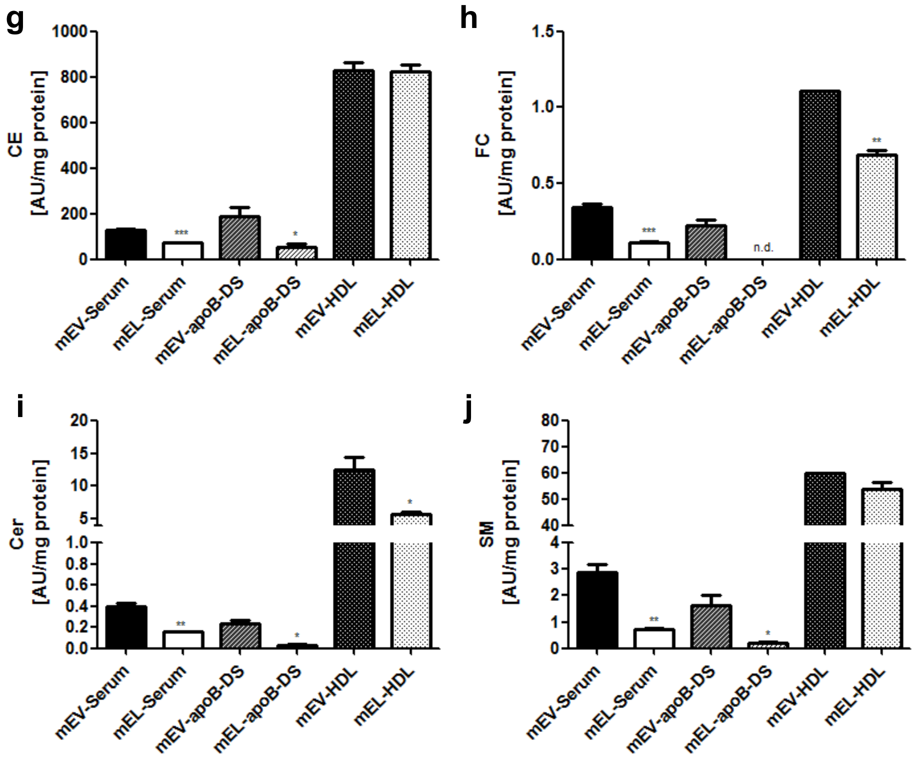

Supplementary Fig. S6 (g-j). EL overexpression in mice alters lipid composition of serum, apoB-DS and HDL
Lipids from mEV-serum, mEL-serum, mEV-apoB-DS, mEL-apoB-DS, mEV-HDL and mEL-HDL (corresponding to 300 μg serum or HDL protein) were extracted and (g) CE, (h) FS, (i) Cer, (j) SM were analysed by MS. Results are mean ± SEM of 3 measurements for each serum, apoB-DS and HDL obtained in 2 independent *in vivo* modifications, with 12 EL-Ad- and 4 EV-Ad-transduced mice per each modification. *$P < 0.05$, **$P < 0.01$, **$P < 0.001$.

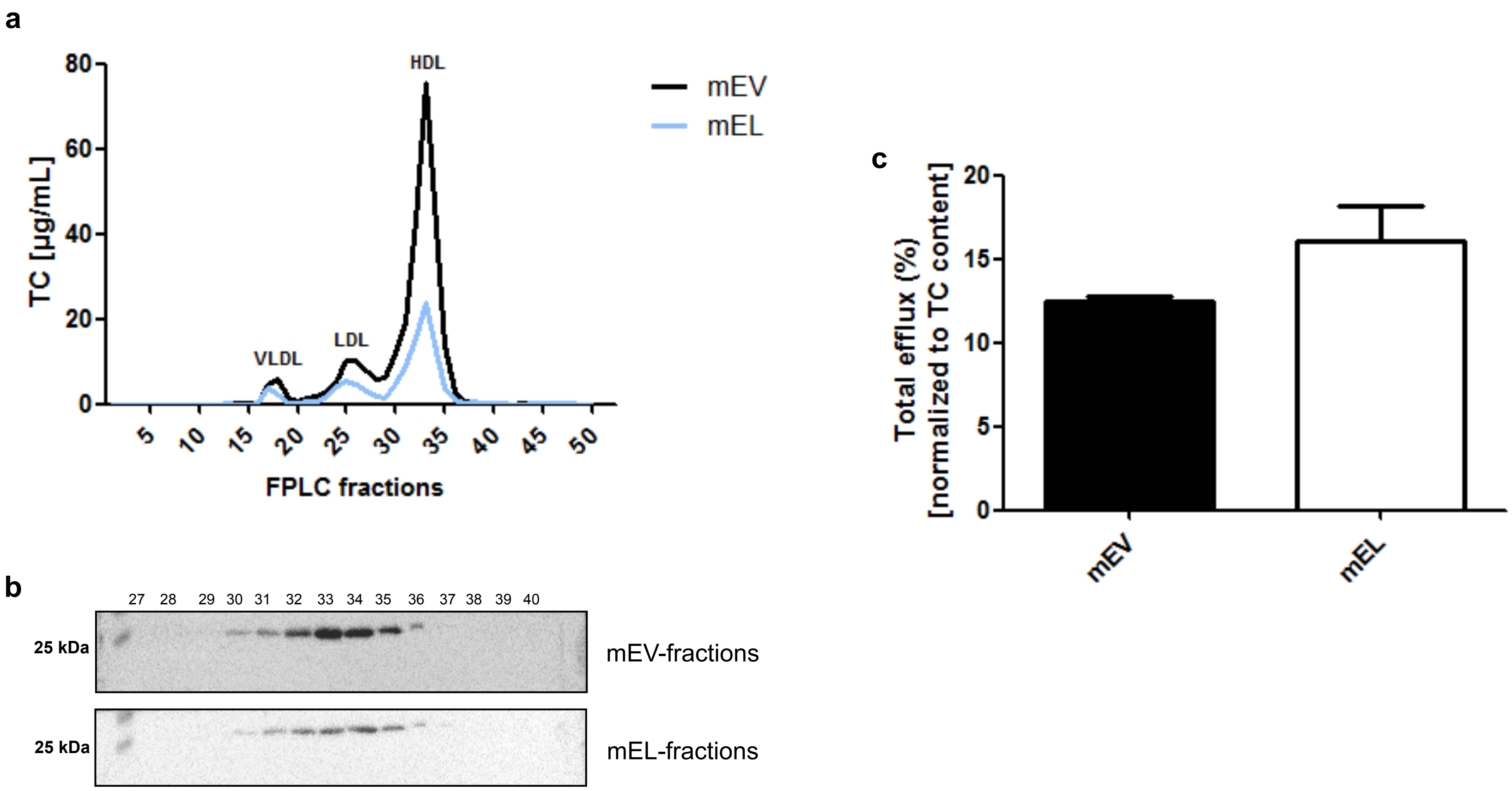

Supplementary Fig. S7. Western blot and CEC of the FPLC fractions of mEV-serum and mEL-serum following EL overexpression in mice

a) FPLC fractions 27 – 40 were analysed by b) SDS-PAGE and apoA-I Western blot. c) Total CEC of pooled fractions 27 - 40 was measured in J774 macrophages following stimulation of ABCA1 expression and normalized to TC content of the pooled FPLC fractions. Protein size annotations refer to protein marker bands on the membranes. Results are mean ± SEM of 2 *in vivo* modifications, with 12 EL-Ad and 4 EV-Ad -transduced mice per each modification. Cholesterol efflux capacity of pooled fractions of each modification was measured twice in duplicates and analysed by unpaired t-test.

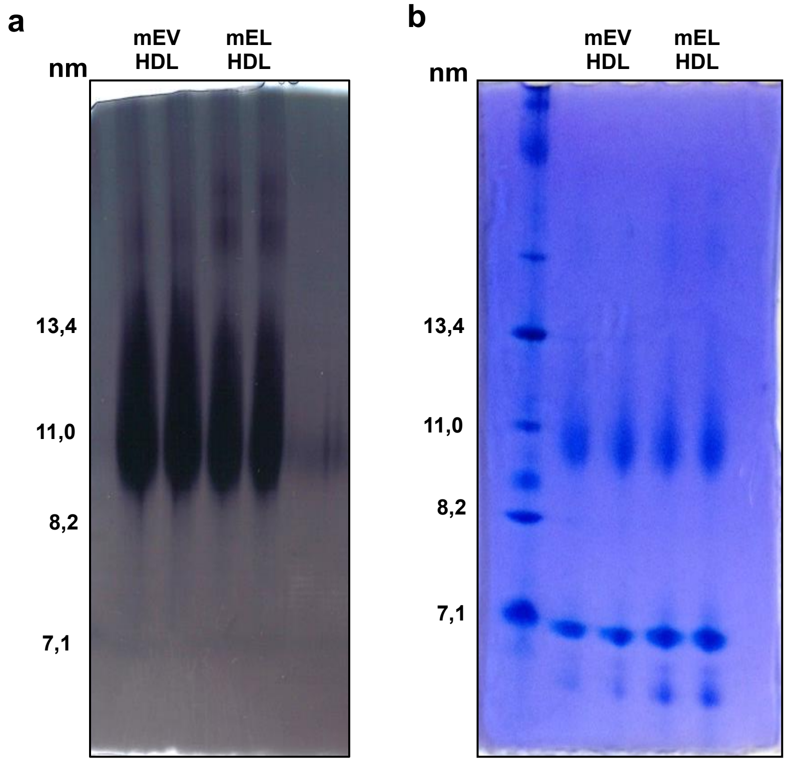

Supplementary Fig. S8. HDL size is unaltered upon overexpression of human EL in mice

Aliquots (10 *μ*g HDL-protein) of mouse (m) EV-HDL and mEL-HDL were electrophoresed on 4-16% non-denaturing polyacrylamide gel followed by Sudan black (a) or Coomassie staining (b). Protein size annotations refer to protein marker bands on the membranes. Results are representative of 2 different *in vivo* modifications with 12 EL-Ad and 4 EV-Ad - transduced mice per each modification.

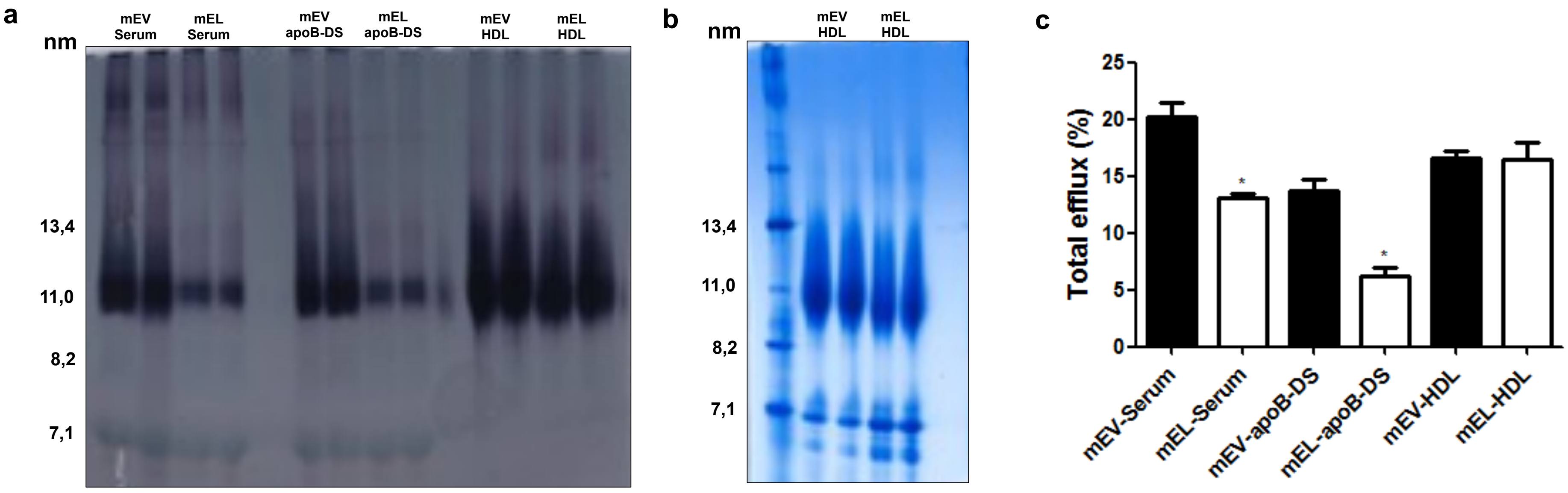

Supplementary Fig. S9. Impact of mouse EL overexpression in mice on HDL serum levels, HDL size and CEC of serum, apoB-DS and isolated HDL

a) Sudan black staining following non-denaturing gradient (4 – 16%) gel electrophoresis of serum, apoB-DS and isolated HDL. b) Coomassie staining of HDL electrophoresed on non-denaturing gradient gel. c) Total CEC measured in J774 macrophages following induction of ABCA1 expression. Results are mean ± SEM of 2 independent *in vivo* modifications, with 12 mEL-Ad and 4 EV-Ad -transduced mice per each modification. Cholesterol efflux capacity of the modified serum, apoB-DS and HDL from each *in vivo* modification was measured twice in duplicates $^*P < 0.05$.

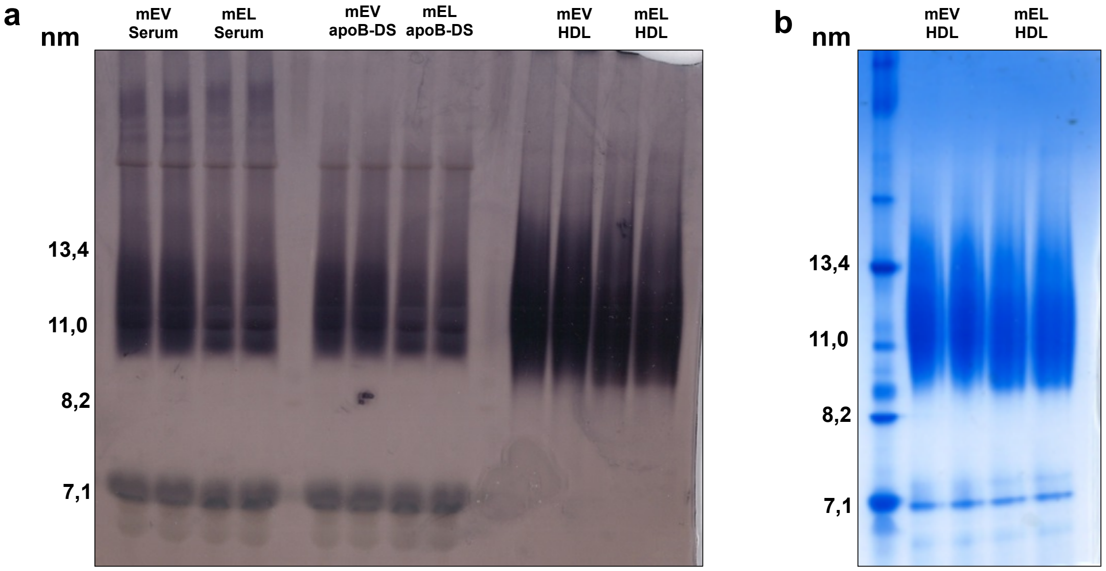

Supplementary Fig. S10. HDL size is unaltered upon EL modification of mouse serum *in vitro*
Aliquots of mEV-serum or mEL-serum (1.5 μL), mEV-apoB-DS or mEL-apoB-DS (2 μL) and mEV-HDL or mEL-HDL (10 μg protein) were electrophoresed on 4-16% non-denaturing polyacrylamide gels followed by Sudan black (a) and Coomassie staining (b). Protein size annotations refer to protein marker bands on the membranes. Results are representative of 2 different modifications of the mouse pool-serum.

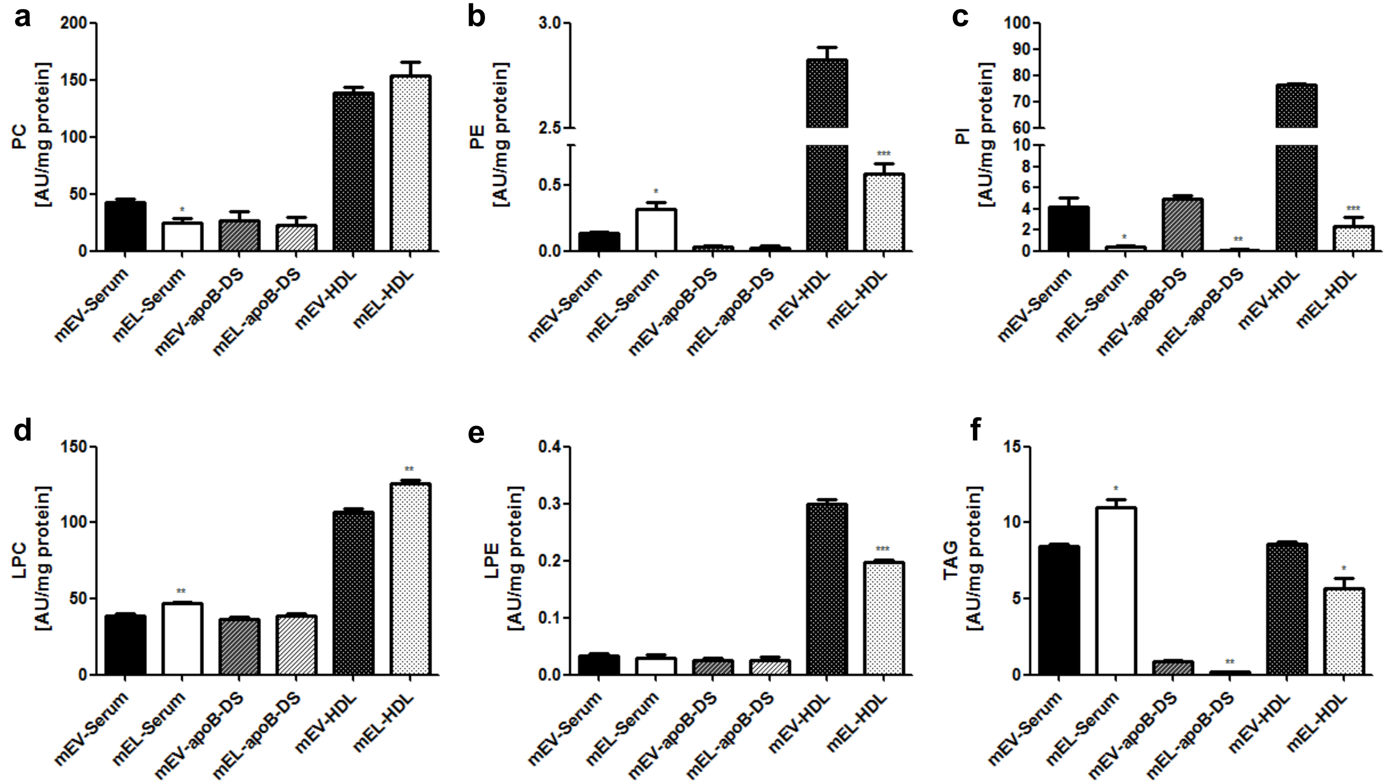

Supplementary Fig. S11 (a-f). Lipid composition of mEL-serum, mEL-apoB-DS, mEL-HDL and respective mEV-controls, following *in vitro* modification of mouse serum with human EL

Lipids from mEV-serum, mEL-serum, mEV-apoB-DS, mEL-apoB-DS, mEV-HDL and mEL-HDL (corresponding to 300 μg serum or HDL protein) were extracted and (a) PC, (b) PE, (c) PI, (d) LPC, (e) LPE, (f) TAG were analysed by MS. Results are mean ± SEM of 3 measurements for each serum, apoB-DS and HDL obtained in 2 independent *in vitro* modifications of pooled mouse serum. *$P < 0.05$, **$P < 0.01$, ***$P < 0.001$.

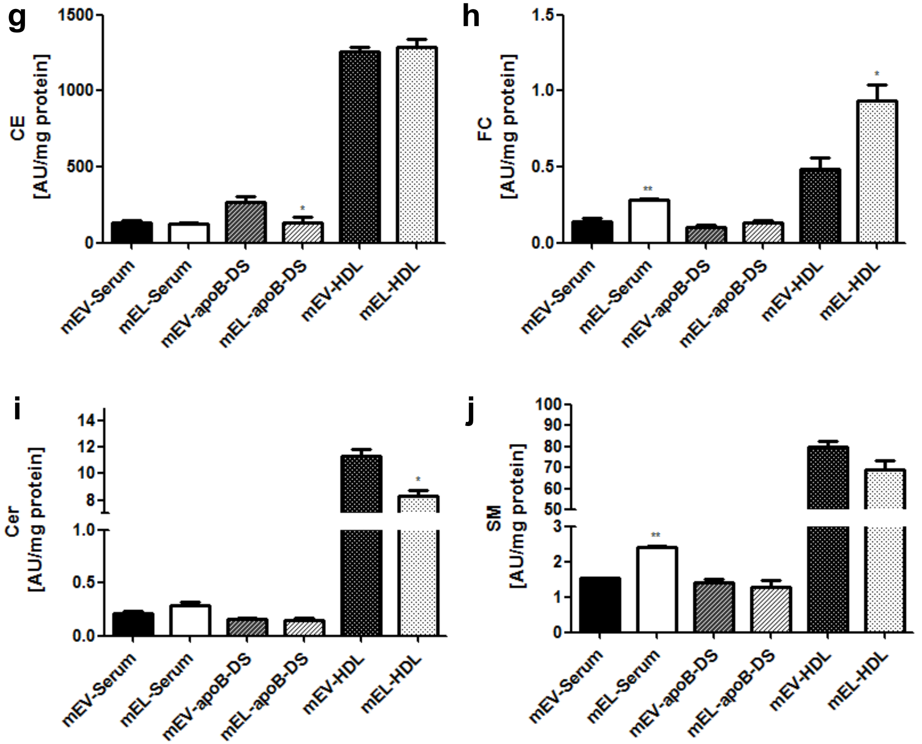

Supplementary Fig. S11 (g-j). Lipid composition of mEL-serum, mEL-apoB-DS, mEL-HDL and respective mEV-controls, following *in vitro* modification of mouse serum with human EL

Lipids from mEV-serum, mEL-serum, mEV-apoB-DS, mEL-apoB-DS, mEV-HDL and mEL-HDL (corresponding to 300 μg serum or HDL protein) were extracted and (g) CE, (h) FS, (i) Cer, (j) SM were analysed by MS. Results are mean ± SEM of 3 measurements for each serum, apoB-DS and HDL obtained in 2 independent *in vitro* modifications of pooled mouse serum. $^{*}P < 0.05$, $^{**}P < 0.01$.

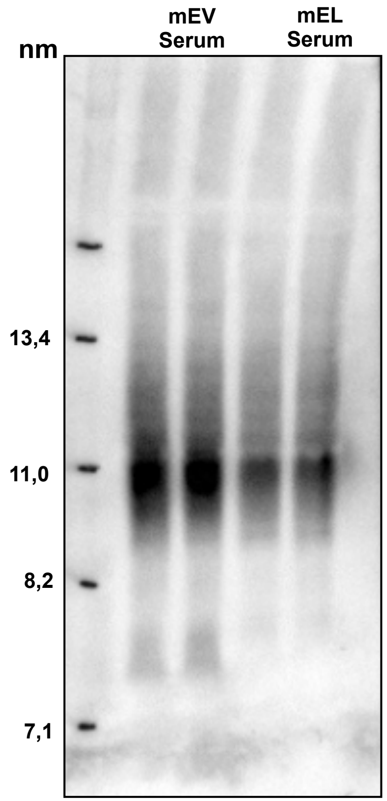

Supplementary Fig. S12. Lipid-free/-poor apoA-I is not detectable in serum of EL-overexpressing mice
Aliquots (1.5 μL) of mEV-serum and mEL-serum were electrophoresed on 4-16% non-denaturing polyacrylamide gels followed by apoA-I Western blot. Protein size annotations refer to protein marker bands on the membranes. Results are representative of 2 different modifications of the mouse pool-serum.

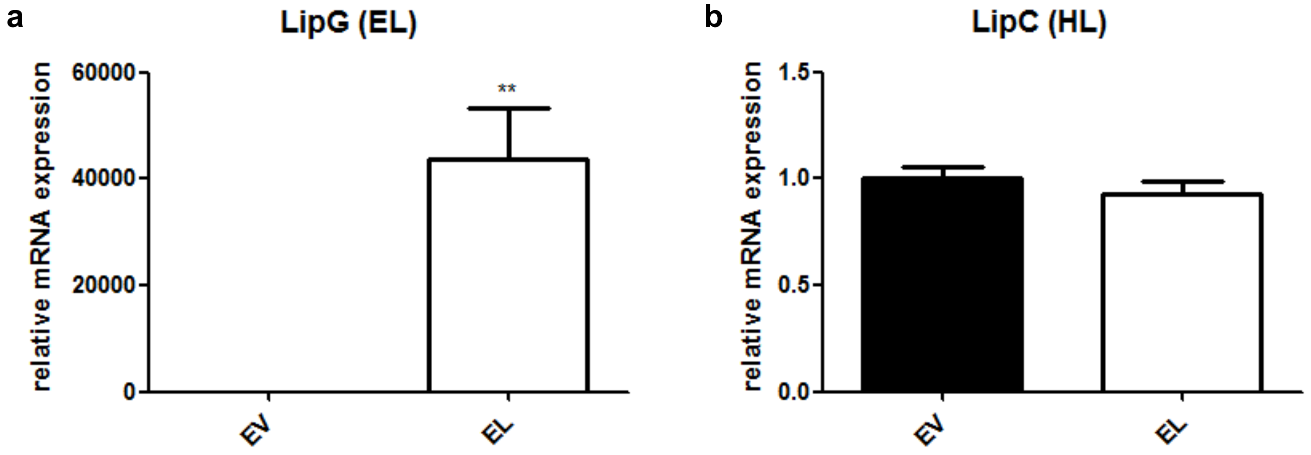

Supplementary Fig. S13. HL mRNA levels are unaltered upon overexpression of human EL in mice
Total RNA was isolated from the liver from 4 EV- and 5 EL-transduced mice, followed by determination of (a) LipG (hEL) and (b) LipC (HL) mRNA expression levels. mRNA expression was analyzed in duplicates by real-time PCR and normalized to cyclophilin A expression as a reference gene. Expression profiles and associated statistical parameters were determined by the $2^{-\Delta\Delta Ct}$ method. $^{**}P < 0.01$.
